# Characterizing the cellular immune response to subretinal AAV gene therapy in the murine retina

**DOI:** 10.1016/j.omtm.2021.05.011

**Published:** 2021-05-29

**Authors:** Laurel C. Chandler, Michelle E. McClements, Imran H. Yusuf, Cristina Martinez-Fernandez de la Camara, Robert E. MacLaren, Kanmin Xue

**Affiliations:** 1 Nuffield Laboratory of Ophthalmology, Nuffield Department of Clinical Neurosciences & NIHR Oxford Biomedical Research Centre, Level 6, West Wing, John Radcliffe Hospital, Headley Way, University of Oxford, Oxford OX3 9DU, UK; 2 Oxford Eye Hospital, Oxford University Hospitals NHS Foundation Trust, Oxford OX3 9DU, UK

**Keywords:** adeno-associated virus, AAV, retinal gene therapy, retinal inflammation, gene therapy-associated uveitis, cellular immune response, flow cytometry, immunity, subretinal injection, microglia

## Abstract

Although adeno-associated viral (AAV) vector-mediated retinal gene therapies have demonstrated efficacy, the mechanisms underlying dose-dependent retinal inflammation remain poorly understood. Here, we present a quantitative analysis of cellular immune response to subretinal AAV gene therapy in mice using multicolor flow cytometry with a panel of key immune cell markers. A significant increase in CD45^+^ retinal leukocytes was detected from day 14 post-subretinal injection of an AAV8 vector (1 × 10^9^ genome copies) encoding green fluorescent protein (GFP) driven by a ubiquitous promoter. These predominantly consisted of infiltrating peripheral leukocytes including macrophages, natural killer cells, CD4 and CD8 T cells, and natural killer T cells; no significant change in resident microglia population was detected. This cellular response was persistent at 28 days and suggestive of type 1 cell-mediated effector immunity. High levels (80%) of GFP fluorescence were found in the microglia, implicating their role in viral antigen presentation and peripheral leukocyte recruitment. When compared against AAV.GFP in paired eyes, an equivalent dose of an otherwise identical vector encoding the human therapeutic transgene Rab-escort protein 1 (*REP1*) elicited a significantly diminished cellular immune response (4.2-fold; p = 0.0221). However, the distribution of immune cell populations remained similar, indicating a common mechanism of AAV-induced immune activation.

## Graphical abstract

**Figure:**
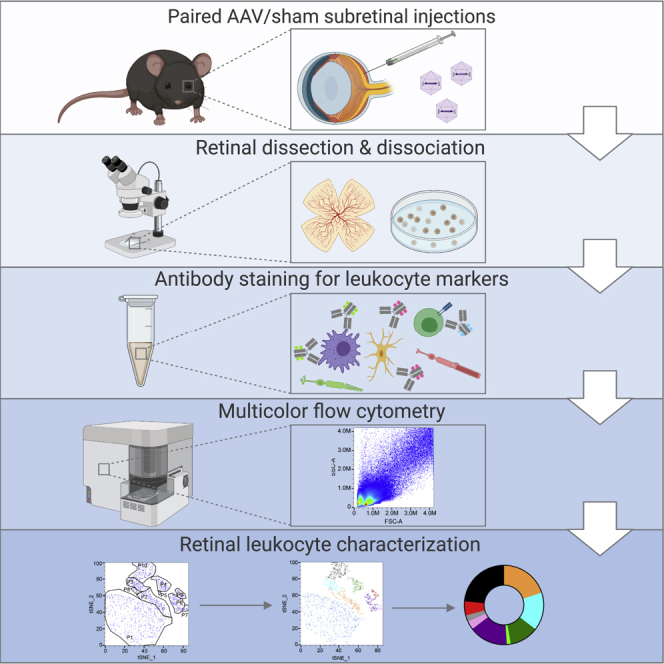

## Teaser

The mechanism of gene therapy-associated uveitis (GTAU) following AAV-mediated retinal gene therapy remains poorly understood. Chandler, Xue, and colleagues utilize multicolor flow cytometry to characterize the cellular immune response to subretinal AAV injections in mice, demonstrating substantial populations of myeloid and T cells from 14 days post-injection.

## Introduction

Adeno-associated viral (AAV) vectors have been widely adopted for gene therapy because of their versatile tissue tropism, ability to stably transduce non-dividing cells without genome integration, and relative low immunogenicity compared to other viral vectors.^1^ AAV-mediated retinal gene augmentation has demonstrated efficacy in clinical trials for inherited retinal dystrophies, including Leber’s congenital amaurosis (LCA),^2^^,^^3^ choroideremia,^4^, ^5^, ^6^ and X-linked retinitis pigmentosa.^7^ The approval of voretigene neparvovec for the treatment of retinal pigment epithelium-specific 65 (*RPE65*)-associated LCA has strengthened the application of AAV-mediated retinal gene therapies as they transition from proof of concept to clinical treatments.

Despite the perceived low immunogenicity of AAV and relative immune privilege of the intraocular environment,^8^ dose-dependent retinal inflammation, or gene therapy-associated uveitis (GTAU), has been observed in many retinal gene therapy trials, which limits the vector dose and therapeutic efficacy. In the phase 1/2 dose-escalation trials for *RPE65*-associated LCA, choroideremia, and retinitis pigmentosa GTPase regulator (*RPGR*)-associated X-linked retinitis pigmentosa, patients receiving higher doses of AAV have presented with various signs of retinal inflammation, including vitritis, retinitis, and choroiditis,^2^^,^^6^^,^^7^ implicating the presence of a cell-mediated immune response. Monitoring of such immune responses to AAV in clinical trial patients has primarily relied on clinical examination and retinal imaging (e.g., optical coherence tomography [OCT]); thus little is known about the mechanisms involved. While patients with signs of intraocular inflammation are generally treated with broad-acting immunosuppression, e.g., systemic or local corticosteroids,^7^ a better understanding of the cellular immune responses involved is critical for designing targeted immune intervention and for improving the safety and visual outcomes following retinal gene therapy.

Under physiological conditions, the blood-retinal barrier, which consists of tight junctions between retinal capillary endothelial cells and between retinal pigment epithelial (RPE) cells, restricts immune surveillance of the retina by the immune system, thus reducing the risk of neuroinflammation.^9^ However, retinal inflammation can induce local expression of pro-inflammatory cytokines and chemokines and can upregulate adhesion molecules on the basal RPE membrane, which leads to the recruitment of leukocytes and breakdown of the blood-retinal barrier.^10^^,^^11^ We and others have shown that *in vivo* AAV subretinal injections induce the expression of pro-inflammatory cytokines and chemokines, including tumor necrosis factor-α (*Tnf-α*), interleukin-1β (*Il-1β*), and C-X-C motif chemokine ligand 10 (*Cxcl10*).^12^, ^13^, ^14^ These molecules function to promote leukocyte recruitment, adhesion, and infiltration through the blood-retinal barrier, ultimately resulting in a breakdown of retinal immune privilege.^15^^,^^16^

Attempts to characterize this cellular response in animal models so far have relied on qualitative immunohistochemical staining of immune cell markers. Preliminary *in vivo* studies have demonstrated increased retinal immunohistochemical staining of the microglia activation marker ionized calcium binding adaptor molecule 1 (Iba1) following subretinal injection of AAV in macaques and mice, suggesting activation of this resident retinal immune cell population.^13^^,^^14^ In addition, immunohistochemical staining in mice for major histocompatibility complex (MHC) class I and II, CD8, and CD20 has suggested active antigen presentation and possible lymphocyte infiltration following subretinal AAV injections.^13^ Flow cytometry provides a sensitive and quantitative means of investigating the cellular response *in vivo* and enables effective discrimination of immune cell populations. Analyses using CD45- and CD11b-specific antibodies have been used to effectively identify microglial and macrophage populations in the normal retina.^17^, ^18^, ^19^ Inclusion of leukocyte-specific markers could provide in-depth understanding of cell-mediated retinal inflammation.

In this study we aimed to investigate the cellular immune response to subretinal AAV-vector mediated gene therapy in wild-type mice, using multicolor flow cytometric analyses to define and quantify distinct immune cell populations. We demonstrated a significantly enhanced cell-mediated response in AAV-injected eyes from day 14 post-injection, including recruitment of several peripheral leukocyte populations that typify a type 1 cell-mediated response. With the use of a *GFP*-expressing vector we detected transgene expression in subsets of these leukocyte populations with significant GFP positivity in resident microglia cells, which suggests a potential role for this population in AAV-mediated immunity. Each of these leukocyte populations was also detected with a vector expressing the human therapeutic transgene Rab-escort protein 1 (*REP1*). Nonetheless, the magnitude of this response was significantly greater in paired eyes injected with equivalent doses of the *GFP*-encoding vector. These findings increase our understanding of the mechanism and timing of AAV-induced cellular immunity.

## Results

### Assessing the effect of AAV gene therapy on the retinal leukocyte population

**Figure 1:**
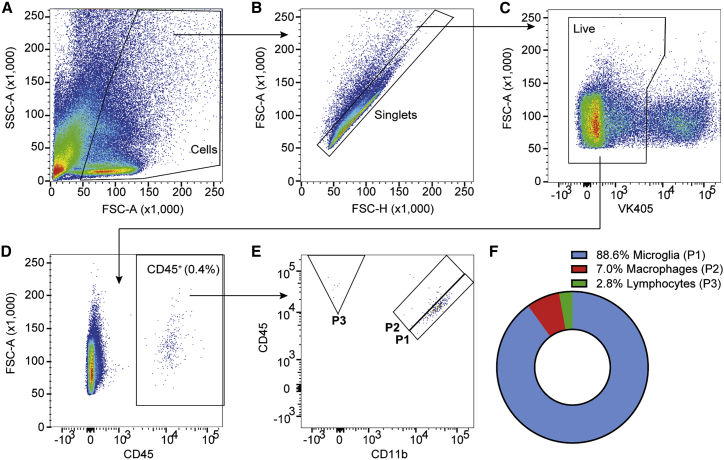
Gating strategy for detection of immune cells in normal adult mouse retina (A–C) The flow cytometry gating strategy for sequential exclusion of debris (A), doublets (B), and dead cells (C) in the dissociated whole retina of a C57BL/6J mouse using the BD LSRFortessa. (D and E) CD45^+^ immune cells were gated for (D) (mean percentage of CD45^+^ cells in the total retina is indicated), and three retinal immune cell populations were identified with differing levels of CD45 and CD11b co-expression (E): (P1; CD45^lo^CD11b^hi^) microglia, (P2; CD45^hi^CD11b^hi^) macrophages, and (P3; CD45^hi^CD11b^−^) lymphocytes. (F) The mean proportions of each of these sub-populations within the CD45^+^ retinal immune cell population; representative of n = 4. SSC-A, side scatter area; FSC-A, forward scatter area; FSC-H, FSC height; VK405, ViaKrome 405.

In order to optimize detection of the resident retinal leukocyte population, whole retinae (excluding the RPE) from 5-week-old wild-type C57BL/6J mice (n = 4) were dissociated, stained with the pan-leukocyte marker CD45 and the myeloid marker CD11b (Table S1), and analyzed by flow cytometry. While CD45^+^ leukocytes constituted a mean 0.4% of all live cells in the normal retina, three distinct CD45^+^ cell populations were consistently identified: 88.6% of the retinal immune cells were CD45^lo^CD11b^hi^ (P1), 7.0% were CD45^hi^CD11b^hi^ (P2), and 2.8% were CD45^hi^CD11b^−^ (P3) (Figure 1). Although both microglia and macrophages are CD11b^+^, they can be distinguished by their level of CD45 expression; microglia typically express low levels of CD45, while macrophages express relatively higher levels of CD45.^20^ The majority of immune cells found in the normal retina were CD45^lo^CD11b^hi^ (P1), suggesting these to be the resident microglial population, while cells defined as CD45^hi^CD11b^hi^ (P2) were likely to be macrophages. In addition to the two CD11b^+^ populations, a CD11b^−^ immune population was also detected (P3). These cells had a low light scatter and hence were predicted to be derived from a lymphoid lineage.

**Figure 2:**
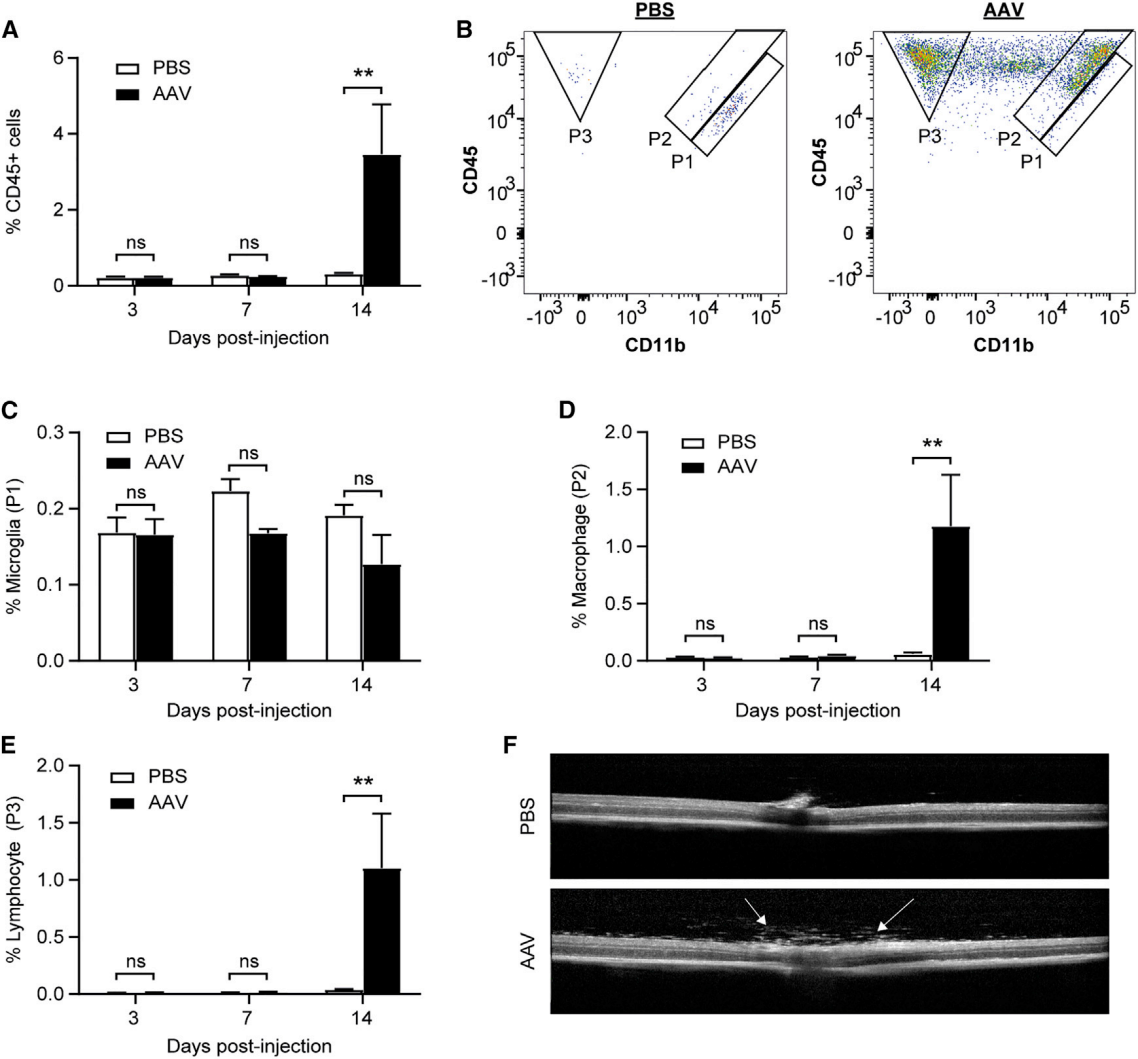
Subretinal AAV gene therapy leads to increases in retinal immune cell populations Wild-type C57BL/6J mice received subretinal injections of PBS or 1 × 10^9^ gc of AAV8(Y733F).CAG.GFP.WPRE in paired eyes. Retinae were harvested for flow cytometric analysis using the BD LSRFortessa at 3, 7, and 14 days post-injection with a stopping gate of 200,000 applied to all events. (A) Percentage of CD45^+^ cells in the retina gated to total live cells. (B) Based on distinct patterns of CD45 and CD11b expression, paired PBS- and AAV-injected retinae from a representative mouse at day 14 post-injection show three immune cell populations, P1, P2, and P3, which are likely to represent resident microglia, macrophages, and lymphocytes, respectively. (C–E) Changes in the percentages of CD45^lo^CD11b^hi^ (P1; microglia) (C), CD45^hi^CD11b^hi^ (P2; macrophages) (D), and CD45^hi^CD11b^−^ (P3; lymphocytes) (E) cells gated to total live cells. ∗∗p ≤ 0.01 (two-way repeated-measures ANOVA with Šidák correction for multiple comparisons) (±SEM, n = 5). (F) Representative SD-OCT images of paired PBS- and AAV-injected eyes at 14 days post-injection; arrows indicate vitreous opacities, suggestive of inflammation.

In order to assess the effects of AAV gene therapy on the retinal immune cell population, wild-type C57BL/6J mice underwent subretinal injections of either PBS or 1 × 10^9^ genome copies (gc) of AAV8(Y733F).CAG.GFP.WPRE in paired eyes. The *GFP* transgene was driven by a ubiquitous cytomegalovirus early enhancer and chicken beta-actin hybrid (CAG) promoter and included a woodchuck hepatitis virus post-transcriptional regulatory element (WPRE), as used in therapeutic vectors for a number of clinical trials. This dose of AAV was selected as it has been shown to be both safe and efficacious in pre-clinical murine studies.^21^^,^^22^ Retinae were harvested at 3, 7, and 14 days post-injection (n = 5 for each time point) and were stained with CD45 and CD11b antibodies prior to flow cytometric analysis. Although no significant difference was seen between paired PBS- and AAV-injected eyes at 3 and 7 days, by 14 days there was a significant increase in the percentage of total CD45^+^ cells in the AAV-injected eyes (two-way ANOVA, p = 0.0038; n = 5) (Figure 2A). Based on co-expression of CD45 and CD11b, three distinct populations of leukocytes (gated as P1, P2, and P3) could be seen in the PBS-injected eyes. In contrast, in AAV-injected eyes the retinal leukocytes spanned a spectrum of CD11b expression levels with cells appearing outside of these three gates (Figure 2B). There was no significant change in the proportion of the CD45^lo^CD11b^hi^ population in the P1 gate at any time point tested (Figure 2C). However, by day 14 post-injection, there was a significant increase in the percentage of CD45^hi^CD11b^hi^ cells within the P2 gate (two-way ANOVA, p = 0.0031; n = 5) (Figure 2D) and CD45^+^CD11b^−^ cells within the P3 gate (p = 0.0066; n = 5) (Figure 2E) in AAV-treated retinae. In addition, *in vivo* spectral domain OCT (SD-OCT) imaging was performed on all mice analyzed by flow cytometry. Vitreous opacities were detected in three of five eyes that received subretinal injections of AAV, which are suggestive of immune cell infiltration (Figure 2F). Moreover, the presence of vitreous opacities appeared to correlate with the highest proportions of viral transduction (as indicated by the percentage of GFP^+^ cells) and immune cell infiltration (CD45^+^ cells) (Table 1; Figure S1).

**Table 1:** The percentage of GFP^+^ and CD45^+^ retinal cells 14 days after subretinal injections of an AAV8(Y733F).CAG.GFP.WPRE vector. Vitreous opacities on SD-OCT images were judged by two independent individuals.

Animal	GFP^+^ cells (%)	CD45^+^ cells (%)	Vitreous opacities
1	28.0	0.3	absent
2	56.7	1.4	absent
3	81.6	2.6	present
4	81.9	6.3	present
5	81.9	6.8	present

**Figure 3:**
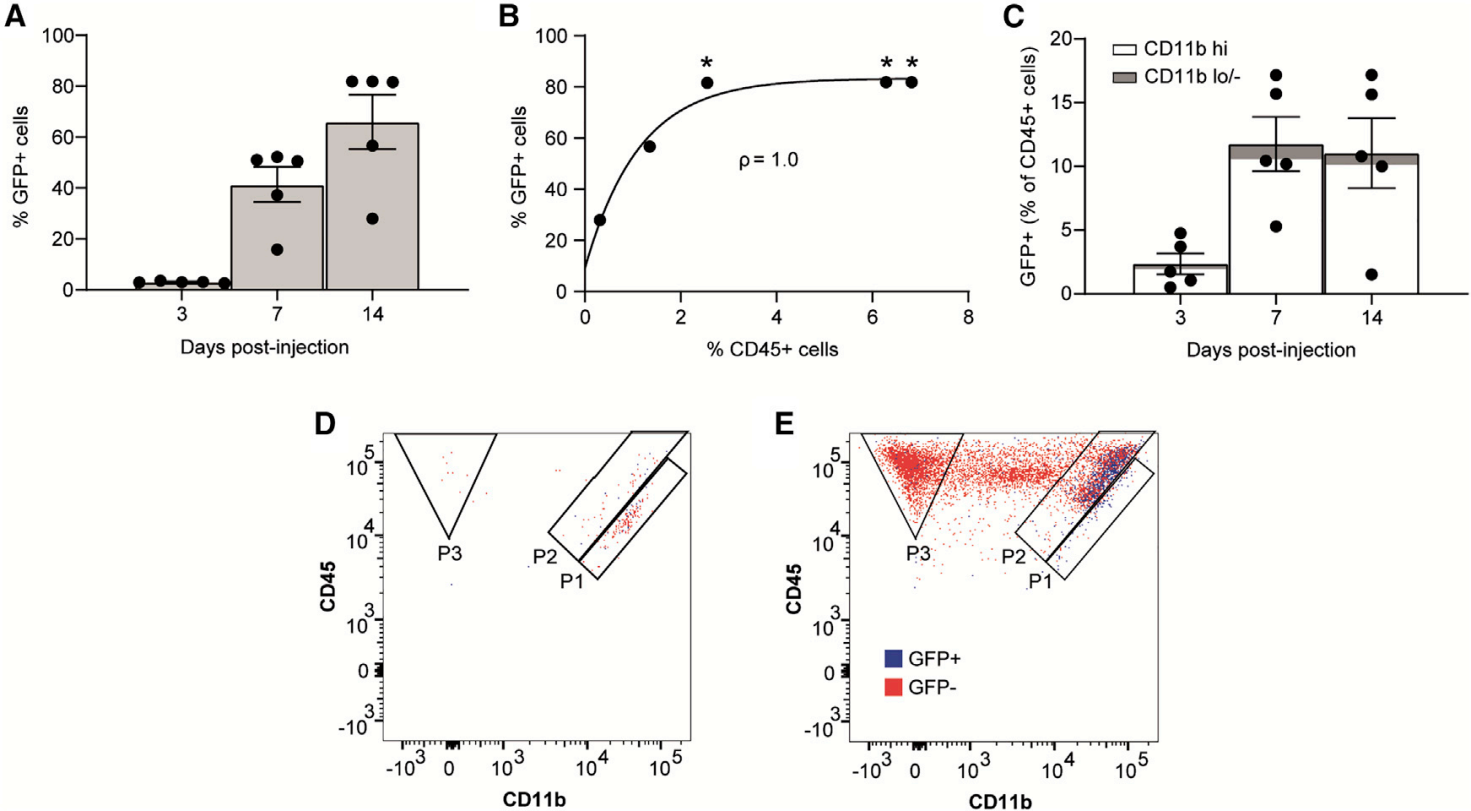
GFP expression following subretinal injection of AAV8(Y733F).CAG.GFP.WPRE in mice Wild-type C57BL/6J mice received a subretinal injection of either AAV8(Y733F).CAG.GFP.WPRE vector or PBS in paired eyes. Retinae were harvested for flow cytometric analysis using the BD LSRFortessa at 3, 7, or 14 days post-injection (n = 5 for each time point). (A) Percentage of GFP^+^ cells in the retina gated to total live cells (±SEM). (B) Nonlinear regression (K > 0) of the correlation between the percentage of GFP^+^ and CD45^+^ at 14 days post-injection. Correlation coefficient (ρ) indicated on graph. Asterisk indicates presence of vitreous opacities on SD-OCT images as per Figure S1. (C) Percentage of GFP^+^ cells within the CD45^+^ population (±SEM); the proportion of this population that is CD11b^hi^ (P1 and P2) compared to the remaining CD11b^lo/−^ cells is indicated. (D and E) Representative plot of an AAV-injected retina 7 (D) and 14 (E) days post-injection with GFP^+^ (blue) and GFP^−^ (red) CD45^+^ immune cells.

GFP fluorescence, a readout for successful AAV transduction, was detected in a mean 3.0% of total retinal cells 3 days post-transduction, which increased to 41.4% by 7 days and 66.0% by 14 days (Figure 3A). A positive correlation of the percentage of GFP^+^ cells to CD45^+^ cells was identified at day 14 post-injection (Spearman’s rank correlation coefficient = 1.0, p = 0.0167) (Figure 3B). Furthermore, a percentage of CD45^+^ cells were also GFP^+^ at all time points post-injection; at day 3 a mean 2.4% of CD45^+^ were GFP^+^, which increased to 11.8% by day 7 and 11.0% by day 14 (Figure 3C). Notably, a mean 81.5% of GFP^+^CD45^+^ cells on day 3, 89.9% on day 7, and 91.6% on day 14 were CD11b^hi^ (Figure 3C), indicating the presence of GFP protein within either microglia or macrophages, which was confirmed by the presence of GFP^+^ events in the P1 gate (Figures 3D and 3E).

### Characterizing the cellular immune response to AAV retinal gene therapy

**Figure 4:**
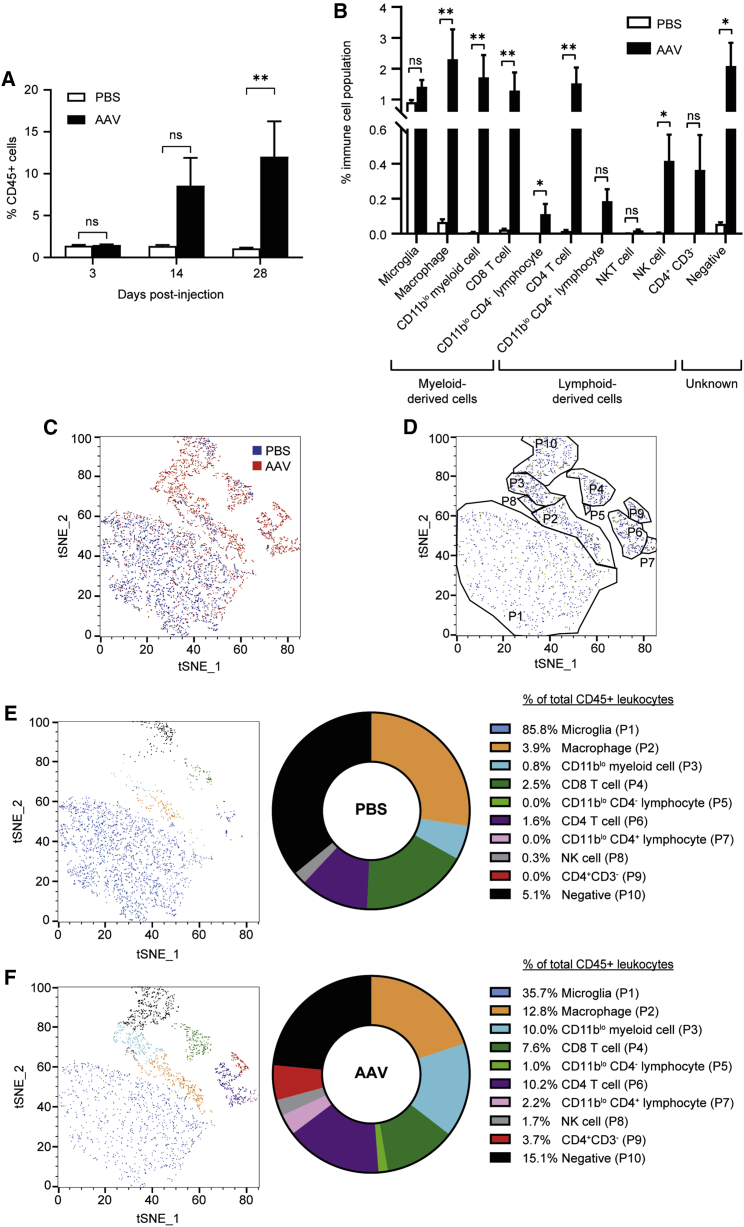
Characterization of the cellular immune response to AAV8.CAG.GFP.WPRE subretinal injections in mice Wild-type C57BL/6J mice received a subretinal injection of either 1 × 10^9^ gc of AAV8.CAG.GFP.WPRE vector or PBS in paired eyes (n = 6 for each time point). Retinae were harvested for multicolor flow cytometric analysis using the Cytek Aurora spectral cytometer at 3, 14, or 28 days post-injection. (A) Percentage of CD45^+^ cells in the retina gated to total cells (two-way repeated-measures ANOVA with Šidák correction). (B) Percentage of CD45^+^ leukocyte populations, including resident microglia, gated to total retinal cells at day 28 post-injection. ∗p ≤ 0.05, ∗∗p ≤ 0.01. Each population was assessed with a ratio paired t test or a Wilcoxon test depending on whether the populations were normally distributed or skewed, respectively (±SEM). (C) Scatterplot of t-SNE dimensional reduction of AAV8.CAG.GFP.WPRE-injected C57BL/6J mice at day 28 post-injection; cells from either PBS- or AAV-injected eyes are colored as blue or red, respectively (2,730 CD45^+^ events per injection material). Data are concatenated from n = 6 per injection material. (D) Gating of different immune cell populations informed by both marker expression and clusters. (E and F) Scatterplot of t-SNE dimensional reduction of PBS (E)- and AAV (F)-injected eyes. Cell populations are colored as indicated in the key with the corresponding percentages of immune cell populations as a fraction of the total retinal CD45^+^ leukocyte population. Corresponding pie charts present the relative proportions of infiltrating leukocyte populations as a fraction of the total CD45^+^ leukocytes. Note that resident microglia have been excluded from the pie charts for clarity, as their numbers remain unchanged as seen in (B).

In order to further characterize the AAV-induced cellular immune response in the retina, multicolor flow cytometric analysis was performed using a panel of leukocyte markers (Table S1) that were optimized in mouse splenocytes to confirm binding specificity and sensitivity (Figure S2). Five-week-old C57BL/6J mice subsequently underwent paired subretinal injections of either PBS or 1 × 10^9^ gc of AAV8.CAG.GFP.WPRE (with a wild-type AAV8 serotype) (n = 6 per time point). Although the AAV8(Y733F) capsid is not known to be more immunogenic, for all subsequent experiments wild-type AAV8 capsid serotype was used in place of the mutant capsid AAV8(Y733F) because of its relevance to retinal gene therapy trials. As seen with injections of AAV8(Y733F).CAG.GFP.WPRE (Figure 2), there was no difference in the percentage of retinal CD45^+^ cells at day 3 post-injection; however, by day 14 there was a substantial increase in this population (Figure 4A). The difference in the percentage of CD45^+^ cells between AAV- and PBS-injected eyes reached statistical significance upon multiple-comparisons testing at day 28 post-injection (two-way ANOVA, p = 0.0088; n = 6). At day 28 a mean 1.1% of total retinal cells were detected as CD45^+^ in PBS-injected samples, which rose to 12.0% in AAV-injected samples.

Retinal cells demonstrate a high level of autofluorescence;^23^^,^^24^ therefore it was necessary to select fluorophores for the flow cytometry panel that minimize spectral overlap with channels containing significant contribution from retinal autofluorescence; this means utilizing many long-emitting fluorochromes, which, because of their inherent properties, can result in greater data spread. A limited antibody panel was initially utilized: CD45, CD11b, CD3, CD4, NK1.1, CD19, and CD11c. With the use of this panel of markers, major immune cell populations were identified within the retina following subretinal injections with AAV8.CAG.GFP.WPRE (Figure S3). At day 28 post-injection, no significant difference in the microglia population was seen between AAV8.CAG.GFP.WPRE- and PBS-injected eyes, which represent ∼1% of the total retinal cells (Figure 4B). However, the majority of other CD45^+^ leukocyte populations were significantly increased in the AAV-treated eyes. These included CD11b^+^ cells of myeloid lineage (including macrophages and an unidentified CD11b^lo^CD11c^−^ myeloid cell) and CD11b^−^ cells of lymphoid lineage (CD8 and CD4 T cells). Although CD11b is typically used as a myeloid cell marker, low-level CD11b expression (CD11b^lo^) was detected in a minority of CD3^+^ lymphocytes, with subsets positive or negative for CD4 (Figure S3). Furthermore, two unknown populations were detected: a CD11b^−^CD3^−^CD4^+^ population (P9), which was not significantly increased at day 28; and a population (P10) that was negative for all immune cell markers except CD45, which was significantly increased in the AAV-treated eyes (Figure 4B). Notably, there was no CD19 or CD11c expression detected in either AAV- or PBS-injected eyes, suggesting an absence of B cells and dendritic cells, respectively. Small populations of natural killer (NK) and natural killer T (NKT) cells were detected in AAV-injected eyes; however, only the NK cell population was significantly increased above sham PBS injections (Figure 4B). The majority sub-populations of NK and NKT cells were found to be CD11b^+^ and CD11b^−^, respectively, which is consistent with mature NK and NKT cell populations (Figure S3). Generally low-level NK1.1 staining was obtained in this experimental cohort (Figure 4) compared with subsequent experiments and the initial optimization in splenocytes (Figure S2). This discrepancy was attributable to degradation of the NK1.1 PE-Cyanine7 tandem-dye antibody aliquot likely due to inadvertent light exposure or poor storage conditions; thus a new aliquot with the same lot number was used in later experiments.

In order to visualize these immune cell populations, t-distributed stochastic neighbor embedding (t-SNE) dimensional reduction was performed on concatenated (pooled) samples from both PBS- and AAV-injected eyes of all animals. A scatterplot of this t-SNE analysis, representing an average distribution of all retinae, indicated the appearance of clusters that were absent in PBS-injected eyes but present in those injected with AAV (Figure 4C). Characterization of these clusters using co-expression of specific markers enabled visualization of these immune cell populations (Figure 4D). In PBS-injected eyes, t-SNE analysis revealed that the majority of immune cells were microglia (Figure 4E). In AAV-injected eyes, significant proportions of the total CD45^+^ immune cells detected in the retinae consisted of leukocytes of both myeloid and lymphoid lineages (Figure 4F). Pie charts are used to visualize the relative proportions of the infiltrating CD45^+^ leukocyte populations. Note that microglia were excluded from the pie charts, as their numbers remained unchanged between PBS- and AAV8.CAG.GFP.WPRE-injected eyes (Figure 4B). Compared with sham PBS-injected eyes, the AAV-injected eyes showed increased populations of macrophages, CD8 and CD4 T cells, an unidentified myeloid population, and a population negative for all markers (Figures 4E and 4F). In addition, the total retinal leukocyte populations following sham injections were very similar to those seen in retinae from age-matched uninjected mice (n = 6) (Figure S4). For instance, 85.8% of the total leukocyte population was microglia in an PBS-injected retina, while in the uninjected retina microglia constituted 89.9% (Figures S4A and S4B). The total CD45^+^ population represented 1.1% and 0.9% of total live retinal cells in PBS-injected and uninjected retina, respectively (Figure S4C).

**Figure 5:**
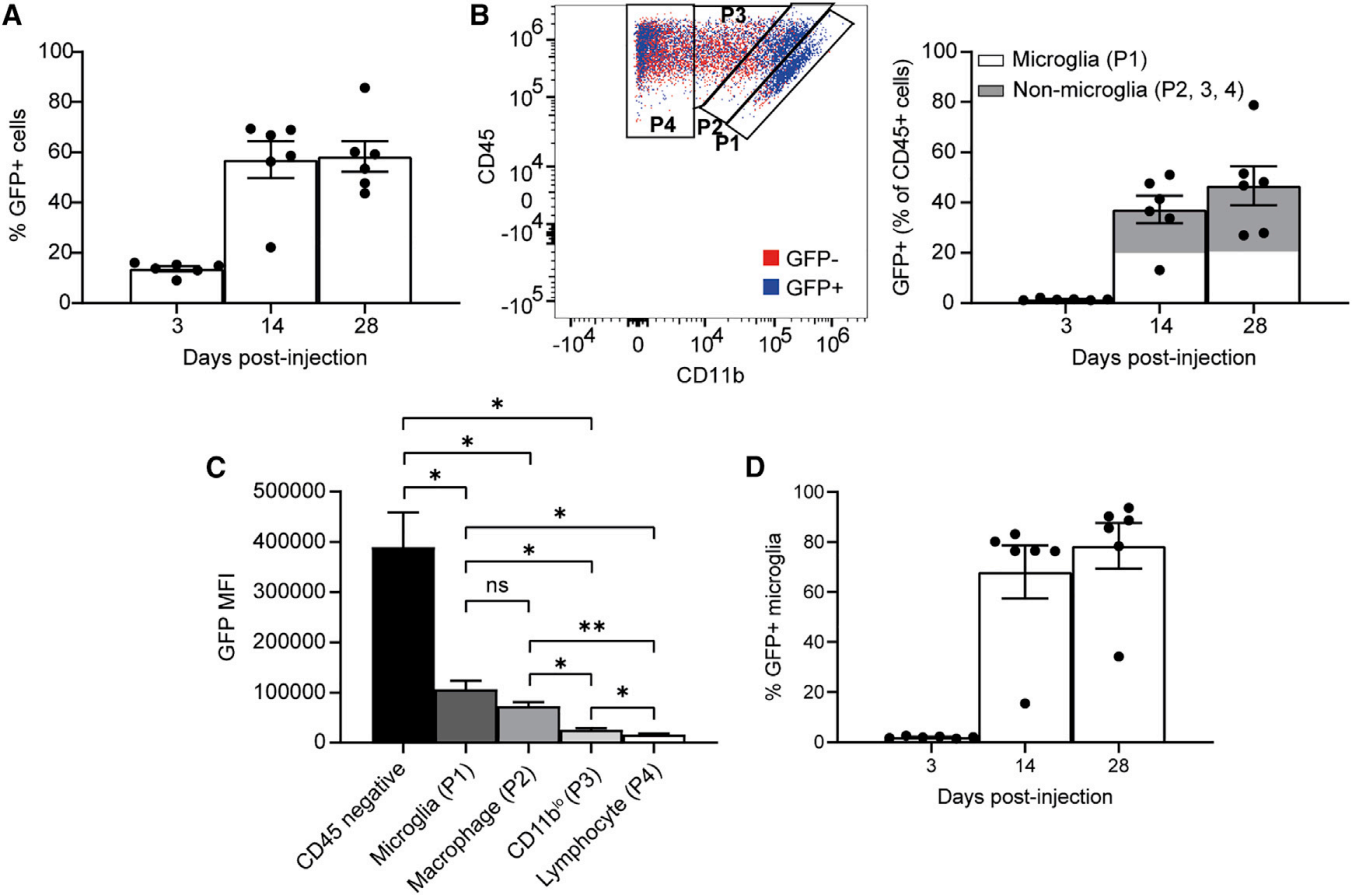
GFP expression following subretinal injections of AAV8.CAG.GFP.WPRE in mice Wild-type C57BL/6J mice received a subretinal injection of either AAV8.CAG.GFP.WPRE or PBS in paired eyes. Retinae were harvested for multicolor flow cytometric analysis using the Cytek Aurora spectral cytometer at 3, 14, or 28 days post-injection. (A) Percentage of total GFP^+^ cells in the retina gated to live cells. (B) Representative plot of an AAV-injected retina 28 days post-injection with GFP^+^ (blue) and GFP^−^ (red) CD45^+^ immune cells. Percentage of GFP^+^ cells within the CD45^+^ population. The proportion of microglia (P1) versus non-microglia cells (P1, P2, and P3) is indicated. (C) GFP MFI of four GFP^+^ immune cell populations as indicated at day 28 post-injection. ∗p ≤ 0.05, ∗∗p ≤ 0.01 (one-way repeated-measures ANOVA with Šidák correction). (D) Percentage of GFP^+^ microglia (P1) gated to total microglia. (±SEM, n = 6).

To further evaluate AAV transduction of retinal immune cells in this injected cohort, which could indicate a potential source of retinal inflammation, the level of GFP transgene expression was assessed. GFP fluorescence was detected in a mean 13.7% of total retinal cells at day 3, 57.1% at day 14, and 58.3% at day 28 post-injection (Figure 5A). Within the CD45^+^ immune cell population a mean 1.5% of cells were GFP^+^ at day 3, 37.2% at day 14, and 46.7% at day 28 post-injection (Figure 5B). AAV transduction of retinal immune cells might be expected to primarily target the resident microglia; while a mean 44.0% of the GFP^+^ leukocytes were microglia, the remaining 56.0% included cells that would not be resident at the time of injection (Figure 5B). However, the GFP median fluorescence index (MFI), used as a readout for the level of transgene expression per cell, was significantly greater in microglia (P1) than CD11b^lo^ cells (P3; one-way ANOVA, p = 0.0192, n = 6) and lymphocytes (P4; p = 0.0132, n = 6). Similarly, the GFP MFI was significantly greater in macrophages (P2) than in P3 (p = 0.0267, n = 6) and P4 (p = 0.007, n = 6) (Figure 5C). Nonetheless, the GFP MFI was significantly higher in total CD45 negative cells than in all leukocyte populations (P1, p = 0.0313; P2, p = 0.0244; P3, p = 0.0197; P4, p = 0.0167; n = 6). A high proportion of microglia were GFP^+^ at days 14 and 28, with means of 68.1% and 78.5%, respectively (Figure 5D).

### Level of cellular immune response may vary with different transgenes

**Figure 6:**
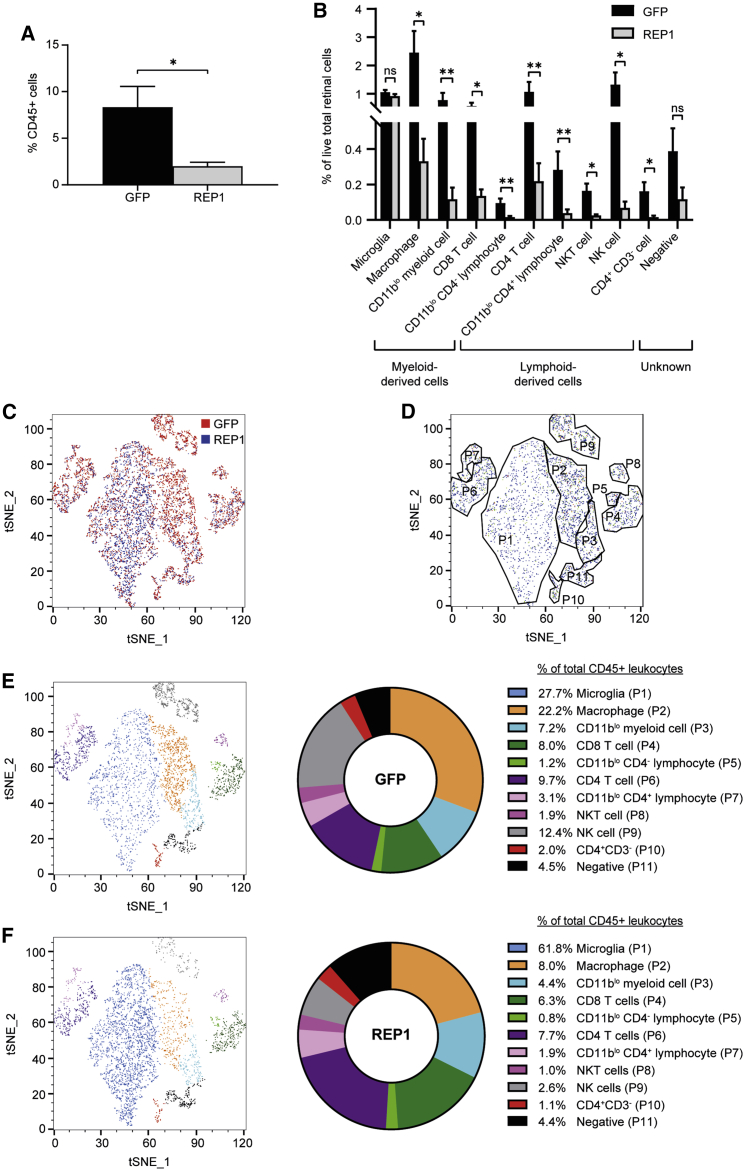
Effect of transgene on the cell-mediated immune response to subretinal AAV gene therapy Wild-type C57BL/6J mice received a subretinal injection of 1 × 10^9^ gc of either AAV8.CAG.GFP.WPRE (GFP) or AAV8.CAG.hREP1.WPRE (REP1) vector in paired eyes (n = 6 per time point). Retinae were harvested for multicolor flow cytometric analysis using the Cytek Aurora spectral cytometer at 14 days post-injection. (A) Percentage of live CD45^+^ cells in the retina gated to total cells (paired t test). (B) Percentage of CD45^+^ leukocyte populations, including resident microglia, gated to total retinal cells. ∗p ≤ 0.05, ∗∗p ≤ 0.01. Each population was assessed with a ratio paired t test or a Wilcoxon test depending on whether the populations were normally distributed or skewed, respectively (±SEM). (C) Scatterplot of t-SNE dimensional reduction of cells from eyes injected with either GFP or REP1, which are colored red or blue, respectively (9,000 CD45^+^ events per injection material). Data are concatenated from n = 6 per injection material. (D) Gating of different immune cell populations informed by both marker expression and clusters. (E and F) Scatterplot of t-SNE dimensional reduction of GFP (E) and REP1 (F) vector-injected eyes. Cell populations are colored as indicated in the key with the corresponding percentages of immune cell populations as a fraction of the total retinal CD45^+^ leukocyte population. Corresponding pie charts present the relative proportions of infiltrating leukocyte populations as a fraction of the total CD45^+^ leukocytes.

To investigate whether variation in the transgene may affect the level of cellular immune response to AAV gene therapy in the retina, subretinal injections of AAV8 vectors that were identical except for the transgene (1 × 10^9^ gc of either AAV8.CAG.GFP.WPRE or AAV8.CAG.hREP1.WPRE) were performed in paired eyes at equivalent doses (n = 6). The expression of both *GFP* and *hREP1* (human *REP1*) transgenes were driven by the ubiquitous CAG promoter and enhanced by WPRE; both vectors were titered simultaneously using the same primer pair. A different AAV2 construct has been used in retinal gene therapy trials to treat choroideremia, a blinding disease caused by loss-of-function mutations in the *CHM* gene that encodes REP1.^25^ The treated retinae were harvested at 14 days post-injection and stained with a panel of immune markers for flow cytometric analysis; CD19 and CD11c antibodies were omitted from these experiments because of lack of staining. The results showed a significant difference in the total percentage of CD45^+^ retinal cells between paired AAV8.GFP (mean 8.3%)- and AAV8.REP1 (mean 2.0%)-injected eyes at 14 days post-injection (paired t test, p = 0.0221, n = 6) (Figure 6A). No difference was seen in the resident microglia population between the GFP and REP1 vector-treated eyes, which remained at ∼1% of all retinal cells (Figure 6B) and was consistent with the previous experiment (Figure 4). However, almost all other infiltrating CD45^+^ leukocyte populations were elevated in the GFP vector-treated retinae, including macrophages, undefined CD11b^lo^CD11c^−^ myeloid cells, CD8 T cells, CD11b^lo^CD4^−^ lymphocytes, CD4 T cells, CD11b^lo^CD4^+^ lymphocytes, NKT cells, NK cells, CD11b^−^CD3^−^CD4^+^ cells, and a leukocyte population negative for all markers except CD45 (Figure 6B; Figure S5). Of note, the overall percentage of CD45^+^ retinal immune cells at day 14 was similar between AAV8.GFP-injected eyes from this cohort and the previous experiment (Figure 4), indicating reproducibility of the immune response (Figure S6).

A t-SNE dimensional reduction was performed on the concatenated samples from both AAV8.GFP- and AAV8.REP1-injected eyes of all animals. A scatterplot of the t-SNE analysis indicated that all the major infiltrating leukocyte clusters were present in eyes injected with both vectors (Figures 6C and 6D). The relative proportions of these leukocyte populations were generally very similar, with the exception of macrophages and NK cells, which were slightly more prevalent in the AAV8.GFP-treated retinae (Figures 6E and 6F).

**Figure 7:**
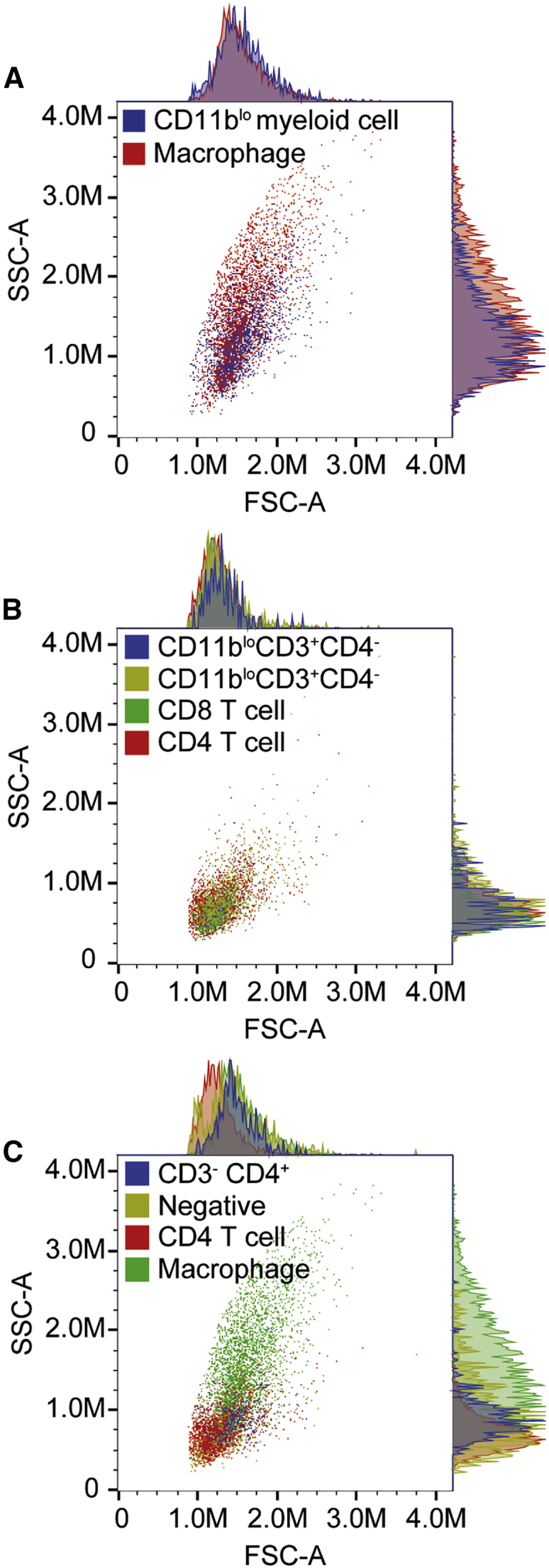
Light scatter profiles of unknown leukocyte populations Representative FSC-A versus SSC-A plots with corresponding histograms of a single C57BL/6J mouse that had received a subretinal injection of 1 × 10^9^ gc of AAV8.CAG.GFP.WPRE. (A–C) Overlay of macrophages and putative myeloid (CD11b^lo^CD11c^−^) cells (A); CD4 T cells, CD8 T cells, CD11b^lo^ CD4 T cells, and CD11b^lo^ CD8 T cells (B); and macrophages, CD4 T cells, immune cells negative for all makers, and CD3^−^CD4^+^ cells (C).

To better characterize the poorly defined leukocyte populations (P3, P5, P7, P10, and P11 in Figure 6), their light scatter patterns were compared against known immune cell phenotypes identified by the antibody panel. Forward scatter (FSC) and side scatter (SSC) are related to cell size and internal complexity, respectively. Myeloid cells are typically higher in FSC and SSC than resting lymphocytes, which is reflected in their scatter profiles. The CD11b^lo^CD11c^−^ putative myeloid population (P3 in Figure 6) had a scatter profile similar to macrophages, suggesting that it was of myeloid origin (Figure 7A). The CD11b^lo^CD3^+^ populations (P5 and P7) were confirmed as lymphocytes on the basis of their lower scatter profile, which was comparable with the scatter of CD11b^−^ T cells detected in this study (Figure 7B). The CD11b^−^CD3^−^CD4^+^ cell population (P10) had a scatter slightly higher than CD4 T cells but significantly less than the macrophages identified in this panel, suggesting that this population was likely derived from a lymphoid lineage and could reflect an activated state (Figure 7C). The CD45^+^ immune population negative for all other markers (P11) had a scatter profile close to that of CD4 T cells, suggesting that it was also of lymphoid lineage (Figure 7C).

## Discussion

Clinical trials of subretinal AAV gene therapy indicate an association between retinal inflammation and increasing vector dose, with most cases of clinically significant inflammation occurring at doses ≥ 10^11^ gc.^26^ However, the presence of pre-existing neutralizing antibodies to AAV does not appear to correlate with a higher risk of retinal inflammation or reduced therapeutic efficacy, provided that the AAV vector is delivered via the subretinal route.^27^, ^28^, ^29^ Our current understanding of the cell-mediated immune response to AAV gene therapy in the retina is primarily based on imaging observations of retinal infiltration and edema in patients and qualitative data (i.e., immunohistochemistry) from large animal studies.^13^ Here we have used sensitive multicolor flow cytometric analysis of whole retinae to quantify and characterize the cell-mediated immune response to AAV8 subretinal injections *in vivo* over time.

The results reveal a significant increase in the number of immune cells within the retina from around day 14 post-injection using an AAV8 serotype vector expressing GFP driven by the ubiquitous CAG promoter. With the use of a panel of immune cell markers, cells of both myeloid and lymphoid lineage, which are typically absent from the normal retina,^30^ were identified, suggesting AAV-mediated recruitment and infiltration of leukocytes. Among the myeloid lineage cells detected, which constituted ∼60% of all immune cells present by day 28, were resident microglia, macrophages, and an unidentified CD11b^lo^CD11c^−^ myeloid population. The light scatter profile of this latter population suggests that they are derived from a myeloid lineage; however, the use of additional markers may aid further identification. Normal retinal tissue contains almost no dendritic cells and only occasional macrophages.^31^ The absence of CD11c^+^ cells found in this study suggests that recruitment of dendritic cells is unlikely to play a significant role in AAV-induced retinal inflammation. The lymphoid lineage cells detected in the retina constituted ∼30% of all immune cells present by day 28, which include CD4 T cells, CD8 T cells, NK cells, and NKT cells. Because of significant background autofluorescence of retinal cells, the number of fluorophores available for use during multicolor flow cytometry was limited; thus a decision was made to indirectly identify CD8 T cells by the expression profile of CD4^−^CD3^+^. NK1.1 staining was significantly better in Figure 6 compared to Figure 4; thus it was likely that some of the NK cells were inadvertently classified as the undefined myeloid and negative populations in Figure 4, while some of the NKT cells were classified as CD8 T cells because of the lack of a CD8 antibody in the panel. A small subset (∼3% of all leukocytes present by day 28) of CD3^+^ cells were detected with relatively low levels of CD11b. Although CD11b is typically used as a myeloid cell marker, the scatter profile of these populations suggested lymphoid origin (Figure 7). While the low CD11b positivity was determined with the use of a “fluorescence minus one” (FMO) control, non-specific CD11b staining of T cell populations cannot be ruled out. Alternatively, CD11b expression has been described as a marker of T cell activation;^32^^,^^33^ thus these immune cells might represent activated effector T cell subsets within inflamed retinae. To further delineate the identity of these T cells, assessment of the expression of other activation markers (e.g., CD69, CD25, and CD70) and pro-inflammatory cytokines may be helpful.^34^

Two unknown immune cell populations were detected: (1) a population negative for the all markers used except CD45 (P10 in Figure 4 and P11 in Figure 6) and (2) a CD11b^−^CD3^−^CD4^+^ population (P9 in Figure 4 and P10 in Figure 6). Both these populations were CD11b^−^ and had small scatter profiles, potentially consistent with lymphoid lineage. We speculate that these two populations might represent subsets of innate lymphoid cells (ILCs), which are negative for the markers typically associated with other immune cell lineages and do not express T cell receptors that induce antigen-specific responses.^35^ ILCs are further divided into three subsets, with group 1 ILCs, which include NK cells and ILC1 cells, playing an important role in mediating a type 1 immune response to viral infections.^35^ The presence of NK cells in the AAV-mediated immune response may suggest that the leukocyte population negative for all markers except CD45, which was significantly increased in AAV-injected eyes, may be ILC1 cells. Tissue-resident ILC1 cells have recently been described in the central nervous system but have not yet been reported in the retina.^36^ Alternatively, this CD45^+^-only population might represent RPE cells that have adhered to the retina during tissue harvesting,^37^ as retinal inflammation might upregulate adhesion molecules in RPE cells, thus causing a higher number of contaminating RPE cells in the retinal preparations. In addition, the use of papain for retinal dissociation might lead to a low level of surface marker loss from the cells, which could potentially account for some of the CD45^+^-only population. Furthermore, the CD11b^−^CD3^−^CD4^+^ cells were detected in AAV vector-treated but not sham-treated retinae (Figure 4). These might represent a small subset of ILCs, called lymphoid tissue-inducer (LTi) cells, which are CD3^−^CD4^+^ and play a role in promoting the survival of T cells that support memory B cells within lymphoid tissue.^38^ However, LTi cells typically interact with T cells in lymphoid tissue and are not known to be present in the retina. Therefore, further characterization is needed to validate the hypothesized identities of these unknown leukocyte populations. As the antibody panel size for multicolor flow cytometry is limited and unmixing of fluorochrome spectra in the autofluorescent retinal tissue is technically challenging, our methods and results provide the basis for further in-depth exploration of the retinal immune cell identities and activation states by single-cell RNA sequencing in the future.

The presence of significant numbers of macrophages, NK cells, and CD8 T cells, and the absence of B cells in the AAV-treated retina, suggests a predominantly type 1 cell-mediated response coordinated by CD4 T_H_1 cells.^39^ This is further supported by the expression of classical type 1 cytokines (interferon-*γ* [*Ifn-γ*], *Tnf-α* and *Cxcl10*) in AAV-treated retina *in vivo* in our previous study^12^ and is consistent with immunohistochemical findings and gene expression analysis in the macaque retina after high-dose subretinal AAV injection.^13^ Since the coordinated response of these leukocytes could detect and destroy the transgene-expressing retinal cells, suppression of this cytotoxic immune response, presenting as either clinical or subclinical retinal inflammation, will be critical for optimizing visual outcomes in retinal gene therapy. Many current retinal gene therapy trials adopt a standardized immunosuppression protocol up to 21 days post-treatment, which may ameliorate this immune response in humans.^6^^,^^7^^,^^40^

We previously demonstrated the upregulation of intracellular innate immune sensors (including Toll-like receptor 9 [*Tlr9*], cyclic GMP AMP synthase [*Cgas*], retinoic acid-inducible gene I [*Rig-I*], tripartite motif containing protein 21 [*Trim21*)], and apolipoprotein B editing complex 3 [*Apobec3*]), effectors (stimulator of interferon genes [*Sting*]), and type 1 interferon response genes in the retina from day 7 post-subretinal AAV gene therapy.^12^ In this study, significant infiltration of leukocytes into the retina was not detected until 14 days, thus suggesting that expression of these innate immune factors likely originates from resident retinal cells. Microglia are the chief resident immune cells of the retina and are capable of inducing pro-inflammatory cytokine expression through TLR9- and TLR2-mediated responses,^41^ which have been demonstrated to detect AAV genomes and capsids, respectively.^42^, ^43^, ^44^ We found a high proportion of retinal immune cells, in particular microglia and macrophages, to be GFP positive, suggesting successful transduction by the AAV8 vector. AAV particles within resident microglia, which can function as antigen-presenting cells and produce pro-inflammatory cytokines,^45^ may be a key source for the recruitment of adaptive immunity. However, it has been previously suggested that microglia are difficult to transduce, with one group detecting no transgene expression by immunohistochemistry following transduction of primary murine microglia *in vitro* with serotypes AAV1–9.^46^ A recent *in vivo* study also demonstrated that, after subretinal AAV delivery, AAV genomes entered microglia at a high rate although their persistence in the nuclei over time was low.^47^ The use of a log unit higher dose of AAV in our experiment and the sensitive quantification of GFP fluorescence spectrum by flow cytometric analysis may explain some of the differences observed between these studies. However, further gene expression analysis in these cells would help to confirm AAV transduction of microglia. The significantly diminished level of GFP expression in all leukocyte populations may support the low level of microglial transduction observed in other studies, which may only be detectable with sensitive flow cytometric analysis. It is conceivable that microglia may have attained GFP protein through phagocytosis of other transduced cells such as photoreceptors, although we would expect this phenomenon to be transient rather than sustained from 14 to 28 days post-injection. Our finding is potentially consistent with recently published data demonstrating AAV8 viral entry into retinal microglia with signal amplification by exchange reaction fluorescence *in situ* hybridization (SABER-FISH).^47^ Interestingly, infiltrating leukocytes not present at the time of subretinal injection were also sometimes weakly GFP positive. This may be the result of retained viable AAV particles in the retina, which have been described in the retina of dogs and primates up to 6 years after gene therapy.^48^ Alternatively, the low GFP intensity (MFI) observed in lymphocytes compared with microglia and macrophages could suggest non-specific binding of GFP protein from lysed photoreceptor cells. These low-level GFP^+^ infiltrating leukocytes were not detected in the earlier experiment performed with the BD LSRFortessa flow cytometer but were detected in the later experiment performed with the Cytek Aurora spectral analyzer, which provides greater sensitivity. The level of GFP expression was also demonstrated to correlate with the percentage of CD45^+^ cells; although only animals with good injections were retained for analysis, one reason for this variability may be technical variation in the subretinal injection. The level of AAV transduction could relate to the size of the subretinal bleb, which may in turn correlate with the level of immune response. Furthermore, the AAV8(Y733F) mutant capsid vector used in the preliminary experiments has been demonstrated to show enhanced transduction due to avoidance of proteasomal degradation. Although increased retinal transduction may induce a greater inflammatory reaction, the differences in flow cytometers used between the experiments with the mutant capsid and wild-type AAV8 vector makes direct comparison difficult.

The overall number of CD45^+^ cells as a proportion of all retinal cells following treatment with the AAV8.GFP vector was reproducible across two independent cohorts of mice, thus validating the quantitative results. On this basis, direct comparison between otherwise identical AAV8 vectors carrying either a *GFP* or *REP1* transgene showed that, by day 14, the latter elicited a significantly reduced cell-mediated immune response (4.2-fold less; p = 0.0221) in the retina at an equivalent vector dose. Although these differences were seen evenly distributed across all infiltrating leukocyte populations, the proportion of resident microglia as a percentage of total retinal cells was similar in the AAV8.GFP- and AAV8.REP1-treated retinae (at ∼1% of all retinal cells). However, although the co-expression of CD45 and CD11b has effectively been used to distinguish microglia and macrophages,^17^, ^18^, ^19^, ^20^^,^^49^ microglia can increase CD45 expression following activation, which may limit the resolution between these two populations in the inflamed retina.^50^^,^^51^ The use of fate-mapping to selectively label microglia could help to definitively identify these cells and analyze their role in response to AAV retinal gene therapy.^19^^,^^51^ With t-SNE analysis, the relative proportions of the infiltrating leukocyte populations can be seen to be relatively conserved between GFP and REP1 vector-injected eyes, with the exception of NK cells and macrophages, which were overrepresented in the GFP vector-treated eyes. Taken together, these findings suggest that the mechanisms of the cell-mediated immune response to the GFP and REP1 vectors were similar, albeit the latter was perhaps occurring at a “subclinical” level. As the capsid and regulatory features of these two vectors were identical, the increased cellular immune response to the GFP vector may be, at least in part, directed toward the *GFP* transgene product. GFP represents a novel antigen in the mouse retina with the potential to trigger an adaptive cytotoxic immune response.^52^ In contrast, while human REP1 is also an exogenous protein, it may be sufficiently similar to murine REP1 as to be better tolerated. The other factor to consider is that, while the GFP and REP1 vectors were both titrated by standardized quantitative polymerase chain reaction (qPCR) and purified to be free of toxic excipients (Figure S7), differences in the level of transduction and protein expression *in vivo* are difficult to control and could potentially affect the scale of any innate immune response. Furthermore, other variables such as the full to empty capsid ratios, reverse packaging rates, or presence of immunogenic components in the GFP DNA sequence (e.g., unmethylated CpGs) may also contribute to the differential immune response between these vectors. Moreover, the AAV8.CAG.GFP.WPRE vector was manufactured with a triple transfection method, while the AAV8.CAG.REP1.WPRE was created with a double transfection method with a combined helper and replication plasmid; this may contribute to some variation in vector packaging efficiency. Finally, prenylation of Rac1 GTPases has been shown to restrain innate immune responses in macrophages,^53^ but it is not known whether prenylation of Rab GTPases by REP1 might have any immunomodulatory effect. To more effectively assess the mechanism of this response, the use of a null vector engineered to contain a similar DNA sequence but that generates no transgene product would be of interest.

Overall, we provide quantitative analysis of the cellular immune response to subretinal gene therapy using flow cytometric analysis. This provides significant benefits over current qualitative techniques, enabling assessment of the timing and scale of cellular immunity *in vivo* while providing a comprehensive visualization of all leukocyte populations. A significant number of leukocytes were detected in retinae from 14 days following subretinal injections of AAV8 vectors expressing a *GFP* transgene under control by a ubiquitous promoter. The cellular inflammatory response comprised a range of leukocytes including macrophages, NK cells, CD4 and CD8 T cells, and NKT cells, suggestive of type 1 effector immunity. While an AAV8.REP1 vector was found to induce a significantly lower level of cellular immune response compared with the equivalent GFP vector, similarities in the overall distribution of infiltrating leukocyte populations suggest a common mechanism of immune activation.

## Materials and methods

### Vector design

All AAV8 transgene plasmids were single-stranded AAVs with a ubiquitous CAG promoter and a WPRE enhancer. CAG.GFP.WPRE vectors expressed *GFP*, and the CAG.hREP1.WPRE vector expressed human *REP1*. CAG.GFP.WPRE vectors were packaged in both a mutant capsid AAV8(Y733F) and a wild-type AAV8 capsid; CAG.REP1.WPRE vectors were packaged into a wild-type AAV8 capsid.

### AAV production

HEK293T cells were grown in HYPERFlasks (Scientific Laboratory Supplies, Nottingham, UK) and transfected with polyethylenimine with a total of 500 mg of endotoxin-free plasmid. AAV.GFP vectors were made by triple transfection using the transgene (CAG.GFP.WPRE), helper (pDeltaAdF6), and capsid-specific RepCap plasmids (Addgene, Watertown, MA, USA). The AAV8.hREP1 vector was made by double transfection using the plasmid CAG.hREP1.WPRE and a combined helper and RepCap plasmid (pDP8.ape) (PlasmidFactory, Bielefeld, Germany). Three days post-transfection, the cells were harvested and lysed, and AAV particles were isolated by ultracentrifugation with an iodixanol gradient. AAV preparations were purified with an Amicon Ultra-15 100K filter unit (Merck Millipore, Gillingham, UK) and collected in sterile PBS. The purity of the AAV preparations was determined by EZBlue (Sigma-Aldrich, Gillingham, UK) staining of sodium dodecyl sulfate-polyacrylamide-gel electrophoresis (SDS-PAGE) gels (Figure S7), and viral titers were determined by qPCR with a primer pair directed to the poly(A) tail (Table S2). All AAV preparations were confirmed to have endotoxin levels < 2 EU/mL with the Pierce LAL Chromogenic Endotoxin Quantitation Kit (Thermo Fisher Scientific).

### Mice

Wild-type C57BL/6J mice (Charles River Laboratories, Margate, UK) were maintained by the Biomedical Science Division, University of Oxford, UK. Mice were housed in a 12-h light-dark cycle, with food and water available *ad libitum*. All animal procedures were approved by local and national ethical and legal authorities and undertaken in accordance with the Association for Research in Vision and Ophthalmology guidelines for the humane use of laboratory animals in ophthalmic research. All procedures were performed under general anesthesia.

### AAV subretinal injections

Paired subretinal injections of 1.5 μL of PBS and AAV were undertaken in C57BL/6J mice. Mice were injected with either AAV8(Y733F).CAG.GFP.WPRE, AAV8.CAG.GFP.WPRE, or AAV8.CAG.hREP1.WPRE at 1 × 10^9^ gc per eye. AAV8(Y733F).CAG.GFP.WPRE-injected mice were harvested at days 3, 7, and 14 post-injection after wide-field SD-OCT imaging (Spectralis HRA, Heidelberg Engineering, Heidelberg, Germany) at each time point with a 55 lens; 8 radial sections were taken with a real-time average process of 25 frames. The absence or presence of vitreous deposits was graded by two independent individuals. Animals injected with either PBS or AAV8.CAG.GFP.WPRE were harvested at 3, 14, and 28 days post-injection. Animals undergoing paired injections of AAV8.CAG.GFP.WPRE or AAV8.CAG.REP1.WPRE were harvested at day 14 post-injection.

### Retinal dissociation

Retinae were harvested using blunt dissection so to minimize any RPE cell carryover. Retinal dissociation was performed immediately after dissection with the Papain Dissociation System (Worthington Biochemical Corporation, Lakewood, NJ, USA). Retinae were enzymatically dissociated in 250 μL of papain-DNase solution (20 U/mL papain and 0.005% DNase) for 14 min at 37°C. 500 μL of Earle’s balanced salt solution (EBSS) was added to each sample before centrifugation at 300 × *g* for 5 min. The cell pellet was resuspended in a solution of 450 μL of EBSS, 50 μL of ovomucoid inhibitor (10 mg/mL of ovomucoid and albumin), and 25 μL of DNase (2,000 U/mL). This solution was carefully layered over 500 μL of ovomucoid and centrifuged at 70 × *g* for 5 min. Dissociated retinal cell pellets were resuspended in staining buffer (2% fetal bovine serum, 2 mM ethylenediaminetetraacetic acid [EDTA], and 0.1% sodium azide in PBS) prior to antibody staining.

### Antibody staining for flow cytometry

Dissociated retinal cells from AAV8(Y733F).CAG.GFP.WPRE-injected mice were stained with the viability dye ViaKrome 405 (Beckman Coulter) at 1/1,000 dilution in PBS for 20 min on ice. Cells were subsequently incubated with TruStain FcX PLUS Fc block (2.5 μg/mL) (BioLegend, London, UK) on ice for 10 min. Cells were washed with staining buffer prior to cell surface antigen staining. Retinal cells from AAV8.CAG.GFP.WPRE- and AAV8.CAG.hREP1.WPRE-injected mice were incubated in Fc block immediately after dissociation and were subsequently stained with cell surface markers. Antibody staining was performed in all retinae on ice for 20 min, with samples protected from light. Cells were subsequently washed with staining buffer prior to flow cytometric analysis. Retinal cells from AAV8.CAG.GFP.WPRE- and AAV8.CAG.hREP1.WPRE-injected mice were stained with 4′,6-diamidino-2-phenylindole (DAPI) (BioLegend, London, UK) 5 min prior to flow cytometric analysis.

The antibodies used in this study were as follows: CD45 Brilliant Violet 711 clone 30-F11 (0.5 μg/mL), CD45 PE clone 30-F11 (0.5 μg/mL), CD11b-APC clone M1/70 (0.25 μg/mL), CD3-Alexa Fluor 700 clone 17A2 (5 μg/mL), CD4 Brilliant Violet 785 clone RM4-5 (0.5 μg/mL), CD19 APC/Fire 750 clone 6D5 (2 μg/mL), NK-1.1 PE/Cyanine7 clone PK136 (1 μg/mL), and CD11c PerCP/Cyanine5.5 clone N418 (1 μg/mL) (all from BioLegend, London, UK).

### Flow cytometry

Compensation (for the BD LSRFortessa) and spectral unmixing (for the Cytek Aurora) was performed using single color controls prepared with CompBeads (BD Biosciences), UltraComp eBeads (Invitrogen) (for CD11c clone N418), GFP BrightComp eBeads (Invitrogen), and dead splenocytes that had been heated at 60°C for 20 min. Flow cytometric analysis of AAV8(Y733F).CAG.GFP.WPRE-injected mice was undertaken with LSRFortessa (BD Biosciences, San Jose, CA, USA). Analysis of all AAV8.CAG.GFP.WPRE- and AAV8.CAG.hREP1.WPRE-injected mice was undertaken with the Aurora spectral analyzer (Cytek Biosciences, Fremont, CA, USA). All data analysis was performed using the FlowJo software (v.10.7.1; BD Life Sciences). Detection of immune cell populations was achieved using expression of various markers and the light scatter profile, where cells of lymphoid lineage are typically low and those of myeloid lineage are higher. Gating of populations was achieved using FMO controls.

### t-SNE analysis

All data analysis was performed using the FlowJo software (v.10.7.1; BD Life Sciences). Downsampling was performed on CD45^+^-gated events of all AAV- and PBS-injected eyes at day 28 post-injection to create a subpopulation with a consistent number of events in all samples. The new Downsample gates were concatenated in all AAV- and PBS-injected samples. t-SNE analysis was performed unsupervised on the compensated parameters CD45, CD11b, CD3, and CD4 for Figure 4 and with the additional marker of NK1.1 for Figure 6, with the following default parameters: iterations = 1,000, perplexity = 30, learning rate (Eta) = 378.

### Statistical analysis

All statistical analysis was performed on GraphPad Prism 8.4.3. Datasets were tested for normality with a Shapiro-Wilk test. Data that were normally distributed were analyzed with a parametric test (t test or ANOVA). Data that were skewed were analyzed with a non-parametric test (Wilcoxon signed rank test). Multiple-comparisons corrections were performed with a Šidák’s test for one- and two-way ANOVA. Paired data were handled accordingly. All statistical tests were performed using an alpha level of 0.05 and two-tail p values. The n and p values are indicated in the figure legends where appropriate. If multiple-comparisons testing has been performed then the p value of the respective comparisons test is described in text. Data are presented as mean ± SEM.

## Supplemental information

Document 1. Figures S1–S7 and Tables S1 and S2

Document 2. Article plus supplemental information

## References

[1] Shirley J.L., de Jong Y.P., Terhorst C., Herzog R.W. (2020). Immune Responses to Viral Gene Therapy Vectors. Mol. Ther..

[2] Bainbridge J.W., Mehat M.S., Sundaram V., Robbie S.J., Barker S.E., Ripamonti C., Georgiadis A., Mowat F.M., Beattie S.G., Gardner P.J. (2015). Long-term effect of gene therapy on Leber’s congenital amaurosis. N. Engl. J. Med..

[3] Russell S., Bennett J., Wellman J.A., Chung D.C., Yu Z.F., Tillman A., Wittes J., Pappas J., Elci O., McCague S. (2017). Efficacy and safety of voretigene neparvovec (AAV2-hRPE65v2) in patients with RPE65-mediated inherited retinal dystrophy: a randomised, controlled, open-label, phase 3 trial. Lancet.

[4] MacLaren R.E., Groppe M., Barnard A.R., Cottriall C.L., Tolmachova T., Seymour L., Clark K.R., During M.J., Cremers F.P., Black G.C. (2014). Retinal gene therapy in patients with choroideremia: initial findings from a phase 1/2 clinical trial. Lancet.

[5] Edwards T.L., Jolly J.K., Groppe M., Barnard A.R., Cottriall C.L., Tolmachova T., Black G.C., Webster A.R., Lotery A.J., Holder G.E. (2016). Visual Acuity after Retinal Gene Therapy for Choroideremia. N. Engl. J. Med..

[6] Xue K., Jolly J.K., Barnard A.R., Rudenko A., Salvetti A.P., Patrício M.I., Edwards T.L., Groppe M., Orlans H.O., Tolmachova T. (2018). Beneficial effects on vision in patients undergoing retinal gene therapy for choroideremia. Nat. Med..

[7] Cehajic-Kapetanovic J., Xue K., Martinez-Fernandez de la Camara C., Nanda A., Davies A., Wood L.J., Salvetti A.P., Fischer M.D., Aylward J.W., Barnard A.R. (2020). Initial results from a first-in-human gene therapy trial on X-linked retinitis pigmentosa caused by mutations in RPGR. Nat. Med..

[8] Taylor A.W. (2016). Ocular Immune Privilege and Transplantation. Front. Immunol..

[9] Cunha-Vaz J., Bernardes R., Lobo C. (2011). Blood-retinal barrier. Eur. J. Ophthalmol..

[10] Crane I.J., Wallace C.A., McKillop-Smith S., Forrester J.V. (2000). Control of chemokine production at the blood-retina barrier. Immunology.

[11] Da Cunha A.P., Zhang Q., Prentiss M., Wu X.Q., Kainz V., Xu Y.Y., Vrouvlianis J., Li H., Rangaswamy N., Leehy B. (2018). The Hierarchy of Proinflammatory Cytokines in Ocular Inflammation. Curr. Eye Res..

[12] Chandler L.C., Barnard A.R., Caddy S.L., Patrício M.I., McClements M.E., Fu H., Rada C., MacLaren R.E., Xue K. (2019). Enhancement of Adeno-Associated Virus-Mediated Gene Therapy Using Hydroxychloroquine in Murine and Human Tissues. Mol. Ther. Methods Clin. Dev..

[13] Reichel F.F., Dauletbekov D.L., Klein R., Peters T., Ochakovski G.A., Seitz I.P., Wilhelm B., Ueffing M., Biel M., Wissinger B., RD-CURE Consortium (2017). AAV8 Can Induce Innate and Adaptive Immune Response in the Primate Eye. Mol. Ther..

[14] Xiong W., Wu D.M., Xue Y., Wang S.K., Chung M.J., Ji X., Rana P., Zhao S.R., Mai S., Cepko C.L. (2019). AAV *cis*-regulatory sequences are correlated with ocular toxicity. Proc. Natl. Acad. Sci. USA.

[15] Aveleira C.A., Lin C.M., Abcouwer S.F., Ambrósio A.F., Antonetti D.A. (2010). TNF-α signals through PKCζ/NF-κB to alter the tight junction complex and increase retinal endothelial cell permeability. Diabetes.

[16] Crane I.J., Liversidge J. (2008). Mechanisms of leukocyte migration across the blood-retina barrier. Semin. Immunopathol..

[17] Gregerson D.S., Yang J. (2003). CD45-positive cells of the retina and their responsiveness to in vivo and in vitro treatment with IFN-gamma or anti-CD40. Invest. Ophthalmol. Vis. Sci..

[18] Li Q., Lan X., Han X., Wang J. (2019). Expression of *Tmem119*/*Sall1* and *Ccr2*/*CD69* in FACS-Sorted Microglia- and Monocyte/Macrophage-Enriched Cell Populations After Intracerebral Hemorrhage. Front. Cell. Neurosci..

[19] O’Koren E.G., Mathew R., Saban D.R. (2016). Fate mapping reveals that microglia and recruited monocyte-derived macrophages are definitively distinguishable by phenotype in the retina. Sci. Rep..

[20] Ford A.L., Goodsall A.L., Hickey W.F., Sedgwick J.D. (1995). Normal adult ramified microglia separated from other central nervous system macrophages by flow cytometric sorting. Phenotypic differences defined and direct ex vivo antigen presentation to myelin basic protein-reactive CD4+ T cells compared. J. Immunol..

[21] Tolmachova T., Tolmachov O.E., Barnard A.R., de Silva S.R., Lipinski D.M., Walker N.J., Maclaren R.E., Seabra M.C. (2013). Functional expression of Rab escort protein 1 following AAV2-mediated gene delivery in the retina of choroideremia mice and human cells ex vivo. J. Mol. Med. (Berl.).

[22] Fischer M.D., McClements M.E., Martinez-Fernandez de la Camara C., Bellingrath J.S., Dauletbekov D., Ramsden S.C., Hickey D.G., Barnard A.R., MacLaren R.E. (2017). Codon-Optimized RPGR Improves Stability and Efficacy of AAV8 Gene Therapy in Two Mouse Models of X-Linked Retinitis Pigmentosa. Mol. Ther..

[23] Liyanage S.E., Gardner P.J., Ribeiro J., Cristante E., Sampson R.D., Luhmann U.F., Ali R.R., Bainbridge J.W. (2016). Flow cytometric analysis of inflammatory and resident myeloid populations in mouse ocular inflammatory models. Exp. Eye Res..

[24] Krause T.A., Alex A.F., Engel D.R., Kurts C., Eter N. (2014). VEGF-production by CCR2-dependent macrophages contributes to laser-induced choroidal neovascularization. PLoS ONE.

[25] Patrício M.I., Barnard A.R., Xue K., MacLaren R.E. (2018). Choroideremia: molecular mechanisms and development of AAV gene therapy. Expert Opin. Biol. Ther..

[26] Bucher K., Rodríguez-Bocanegra E., Dauletbekov D., Fischer M.D. (2020). Immune responses to retinal gene therapy using adeno-associated viral vectors - Implications for treatment success and safety. Prog. Retin. Eye Res..

[27] Li Q., Miller R., Han P.Y., Pang J., Dinculescu A., Chiodo V., Hauswirth W.W. (2008). Intraocular route of AAV2 vector administration defines humoral immune response and therapeutic potential. Mol. Vis..

[28] Reichel F.F., Peters T., Wilhelm B., Biel M., Ueffing M., Wissinger B., Bartz-Schmidt K.U., Klein R., Michalakis S., Fischer M.D., RD-CURE Consortium (2018). Humoral Immune Response After Intravitreal But Not After Subretinal AAV8 in Primates and Patients. Invest. Ophthalmol. Vis. Sci..

[29] Bennett J., Wellman J., Marshall K.A., McCague S., Ashtari M., DiStefano-Pappas J., Elci O.U., Chung D.C., Sun J., Wright J.F. (2016). Safety and durability of effect of contralateral-eye administration of AAV2 gene therapy in patients with childhood-onset blindness caused by RPE65 mutations: a follow-on phase 1 trial. Lancet.

[30] McMenamin P.G., Saban D.R., Dando S.J. (2019). Immune cells in the retina and choroid: Two different tissue environments that require different defenses and surveillance. Prog. Retin. Eye Res..

[31] Forrester J.V., Xu H., Kuffová L., Dick A.D., McMenamin P.G. (2010). Dendritic cell physiology and function in the eye. Immunol. Rev..

[32] McFarland H.I., Nahill S.R., Maciaszek J.W., Welsh R.M. (1992). CD11b (Mac-1): a marker for CD8+ cytotoxic T cell activation and memory in virus infection. J. Immunol..

[33] Christensen J.E., Andreasen S.O., Christensen J.P., Thomsen A.R. (2001). CD11b expression as a marker to distinguish between recently activated effector CD8(+) T cells and memory cells. Int. Immunol..

[34] Shipkova M., Wieland E. (2012). Surface markers of lymphocyte activation and markers of cell proliferation. Clin. Chim. Acta.

[35] Walker J.A., Barlow J.L., McKenzie A.N. (2013). Innate lymphoid cells--how did we miss them?. Nat. Rev. Immunol..

[36] Romero-Suárez S., Del Rio Serrato A., Bueno R.J., Brunotte-Strecker D., Stehle C., Figueiredo C.A., Hertwig L., Dunay I.R., Romagnani C., Infante-Duarte C. (2019). The Central Nervous System Contains ILC1s That Differ From NK Cells in the Response to Inflammation. Front. Immunol..

[37] Limb G.A., Cole C.J., Earley O., Hollifield R.D., Russell W., Stanford M.R. (1997). Expression of hematopoietic cell markers by retinal pigment epithelial cells. Curr. Eye Res..

[38] Lane P.J., Gaspal F.M., Kim M.Y. (2005). Two sides of a cellular coin: CD4(+)CD3- cells regulate memory responses and lymph-node organization. Nat. Rev. Immunol..

[39] Annunziato F., Romagnani C., Romagnani S. (2015). The 3 major types of innate and adaptive cell-mediated effector immunity. J. Allergy Clin. Immunol..

[40] Maguire A.M., Simonelli F., Pierce E.A., Pugh E.N., Mingozzi F., Bennicelli J., Banfi S., Marshall K.A., Testa F., Surace E.M. (2008). Safety and efficacy of gene transfer for Leber’s congenital amaurosis. N. Engl. J. Med..

[41] Olson J.K., Miller S.D. (2004). Microglia initiate central nervous system innate and adaptive immune responses through multiple TLRs. J. Immunol..

[42] Zhu J., Huang X., Yang Y. (2009). The TLR9-MyD88 pathway is critical for adaptive immune responses to adeno-associated virus gene therapy vectors in mice. J. Clin. Invest..

[43] Faust S.M., Bell P., Cutler B.J., Ashley S.N., Zhu Y., Rabinowitz J.E., Wilson J.M. (2013). CpG-depleted adeno-associated virus vectors evade immune detection. J. Clin. Invest..

[44] Hösel M., Broxtermann M., Janicki H., Esser K., Arzberger S., Hartmann P., Gillen S., Kleeff J., Stabenow D., Odenthal M. (2012). Toll-like receptor 2-mediated innate immune response in human nonparenchymal liver cells toward adeno-associated viral vectors. Hepatology.

[45] Li L., Eter N., Heiduschka P. (2015). The microglia in healthy and diseased retina. Exp. Eye Res..

[46] Rosario A.M., Cruz P.E., Ceballos-Diaz C., Strickland M.R., Siemienski Z., Pardo M., Schob K.L., Li A., Aslanidi G.V., Srivastava A. (2016). Microglia-specific targeting by novel capsid-modified AAV6 vectors. Mol. Ther. Methods Clin. Dev..

[47] Wang S.K., Lapan S.W., Hong C.M., Krause T.B., Cepko C.L. (2020). *In Situ* Detection of Adeno-associated Viral Vector Genomes with SABER-FISH. Mol. Ther. Methods Clin. Dev..

[48] Stieger K., Schroeder J., Provost N., Mendes-Madeira A., Belbellaa B., Le Meur G., Weber M., Deschamps J.Y., Lorenz B., Moullier P., Rolling F. (2009). Detection of intact rAAV particles up to 6 years after successful gene transfer in the retina of dogs and primates. Mol. Ther..

[49] Dick A.D., Ford A.L., Forrester J.V., Sedgwick J.D. (1995). Flow cytometric identification of a minority population of MHC class II positive cells in the normal rat retina distinct from CD45lowCD11b/c+CD4low parenchymal microglia. Br. J. Ophthalmol..

[50] Greter M., Lelios I., Croxford A.L. (2015). Microglia Versus Myeloid Cell Nomenclature during Brain Inflammation. Front. Immunol..

[51] Plemel J.R., Stratton J.A., Michaels N.J., Rawji K.S., Zhang E., Sinha S., Baaklini C.S., Dong Y., Ho M., Thorburn K. (2020). Microglia response following acute demyelination is heterogeneous and limits infiltrating macrophage dispersion. Sci. Adv..

[52] Khabou H., Cordeau C., Pacot L., Fisson S., Dalkara D. (2018). Dosage Thresholds and Influence of Transgene Cassette in Adeno-Associated Virus-Related Toxicity. Hum. Gene Ther..

[53] Akula M.K., Ibrahim M.X., Ivarsson E.G., Khan O.M., Kumar I.T., Erlandsson M., Karlsson C., Xu X., Brisslert M., Brakebusch C. (2019). Protein prenylation restrains innate immunity by inhibiting Rac1 effector interactions. Nat. Commun..

