# Supplementary material for: Characterizing the cellular immune response to subretinal AAV gene therapy in the murine retina
Source: Mol Ther Methods Clin Dev. 2021 May 29;22:52–65. doi: 10.1016/j.omtm.2021.05.011 (PMC8390455; doi:10.1016/j.omtm.2021.05.011)
Supplement: Document 1. Figures S1–S7 and Tables S1 and S2 [file mmc1.pdf]

OMTM, Volume *22*

Supplemental information

Characterizing the cellular immune response to subretinal AAV gene therapy in the murine retina

Laurel C. Chandler, Michelle E. McClements, Imran H. Yusuf, Cristina Martinez-Fernandez de la Camara, Robert E. MacLaren, and Kanmin Xue

Supplementary material

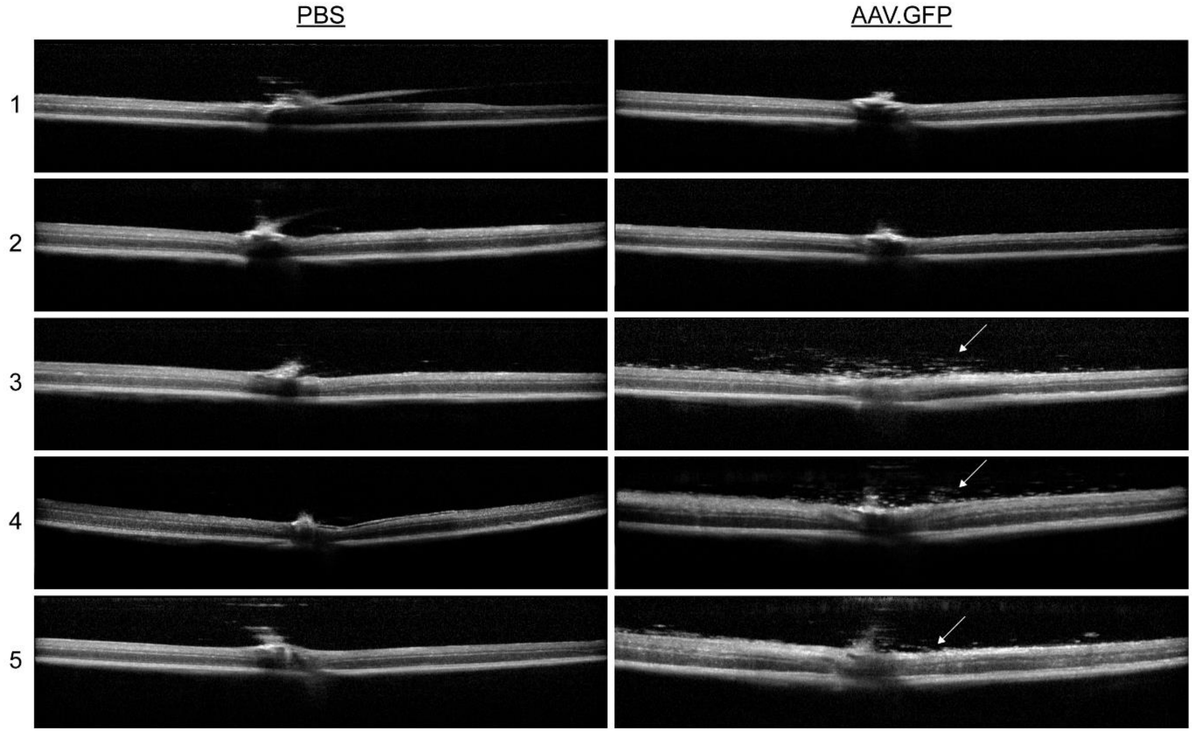

Supplementary figure 1. Vitreous opacities in SD-OCT images of AAV-injected retinae. Wildtype C57BL/6J mice received subretinal injections of PBS or $1x10^9$ gc of AAV8(Y733F).CAG.GFP.WPRE (AAV.GFP) in paired eyes. SD-OCT images were taken 14 days post-injection in five animals (1-5), which correspond to the data presented in Table 1. Arrows indicate vitreous opacities, suggestive of inflammation.

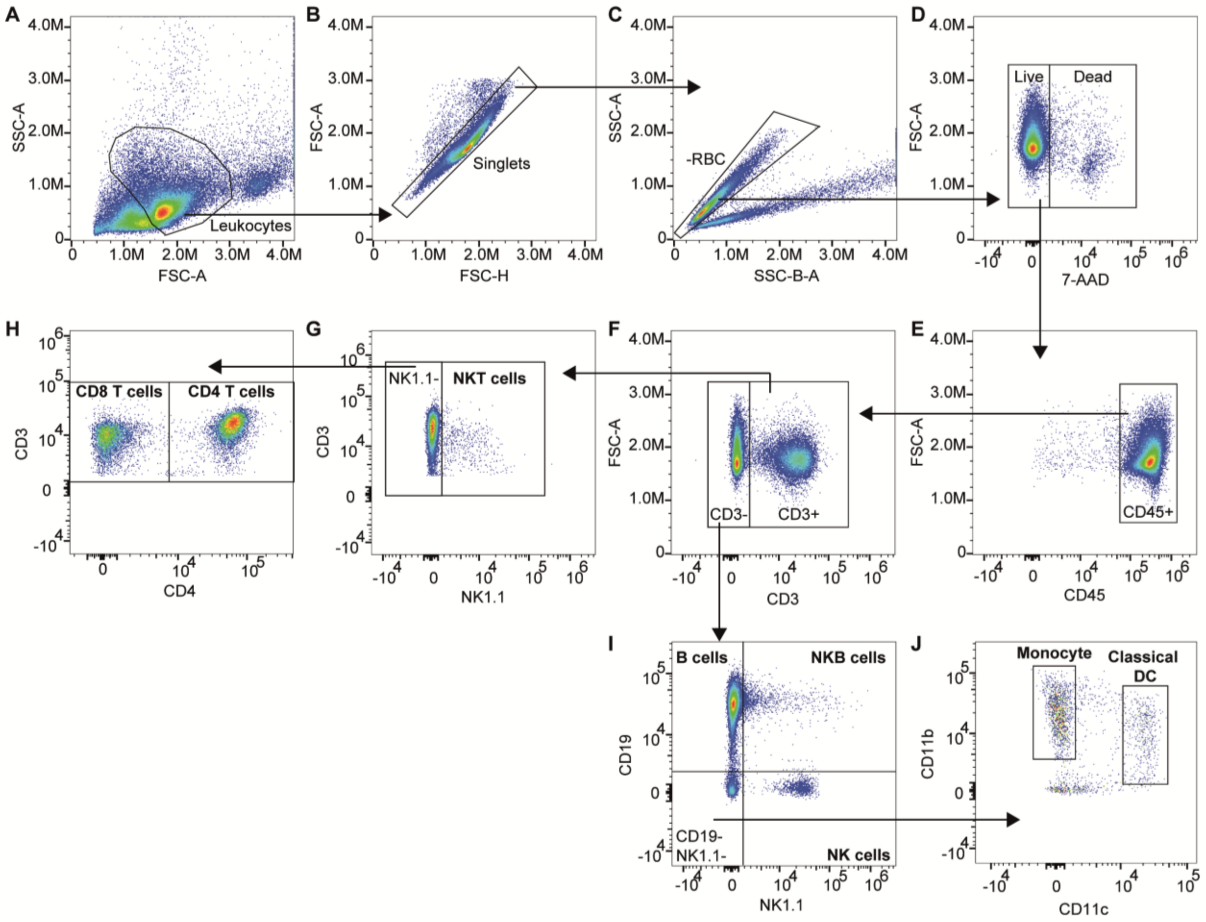

Supplementary figure 2. Gating strategy of leukocyte populations on mouse splenocytes. The flow cytometry gating strategy of C57BL/6J mouse splenocytes assessed using the Cytek Aurora spectral cytometer. The sequential exclusion of (**A**) debris, (**B**) cell doublets, (**C**) red blood cells, and (**D**) dead cells. (**E**) CD45$^+$ immune cells are selected and (**F**) cells were gated according to CD3 expression. (**G**) CD3$^+$NK1.1$^+$ cells were identified as NK T cells and (**H**) NK1.1$^-$ cells were identified as CD3$^+$CD4$^+$NK1.1$^-$ (CD4 T cells) and CD3$^+$CD4$^-$NK1.1$^-$ (CD8 T cells). (**I**) CD3$^-$ populations from panel F were gated as CD19$^+$CD3$^-$NK1.1$^-$ (B cells), CD19$^+$CD3$^-$NK1.1$^+$ (NK B cells), and CD19$^-$CD3$^-$NK1.1$^+$ (NK cells). (**J**) CD19$^-$NK1.1$^-$ cells were gated as CD11b^{hi}CD11c$^-$ (monocytes) and CD11b^{lo}CD11c$^+$ (classical dendritic cells (DC)).

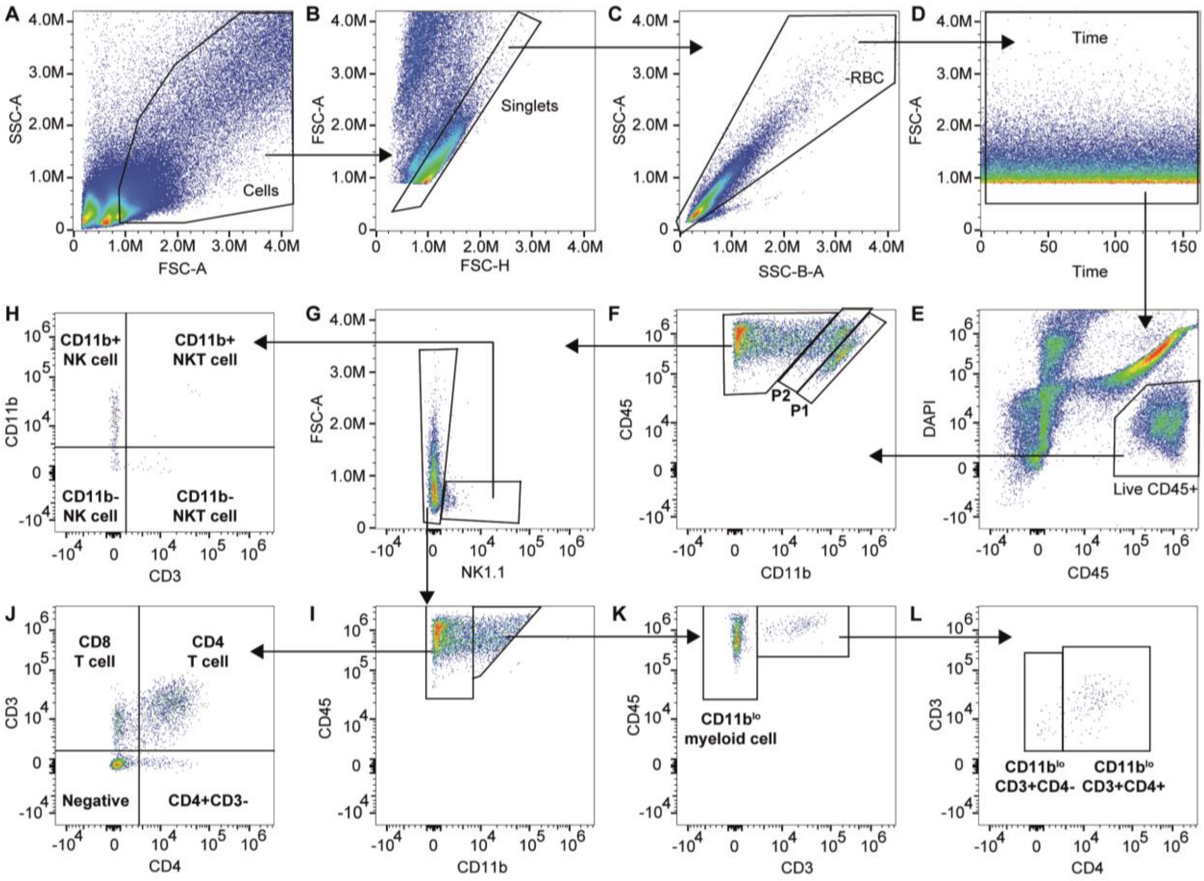

Supplementary figure 3. Gating strategy of an AAV8.CAG.GFP.WPRE-injected mouse retina from Figure 4. The flow cytometry gating strategy of dissociated retinal cells 28 days following injection with an AAV8.CAG.GFP.WPRE vector in C57BL/6J mice from Figure 4 assessed using the Cytek Aurora spectral cytometer. Gating was achieved using fluorescence minus one (FMO) controls. The sequential exclusion of (**A**) debris, (**B**) cell doublets, and (**C**) red blood cells. (**D**) Cells that were not acquired in a continuous flow stream were excluded by applying a time gate. (**E**) Live CD45^{+} leukocytes were gated to exclude dead cells and those not of interest in this study. (**F**) Two CD11b^{hi} expressing populations were identified; microglia (P1) and macrophages (P2). (**G**) NK1.1^{+} cells were identified and (**H**) further delineated as CD11b$^{+/-}$ NK and CD11b$^{+/-}$ CD3^{+} NKT cells. (**I**) CD11b^{-}NK1.1^{-} cells were (**J**) identified as CD3^{+}CD4^{-} (CD8 T cells), CD3^{+}CD4^{+} (CD4 T cells), CD3^{-} CD4^{+}, and CD3^{-}CD4^{-} (negative). CD11b^{lo}NK1.1^{-} cells were identified as (**K**) CD3^{-} myeloid cells and (**L**) CD3^{+} cells, with the populations CD11b^{lo}CD3^{+}CD4^{-} or CD11b^{lo}CD3^{+}CD4^{+}.

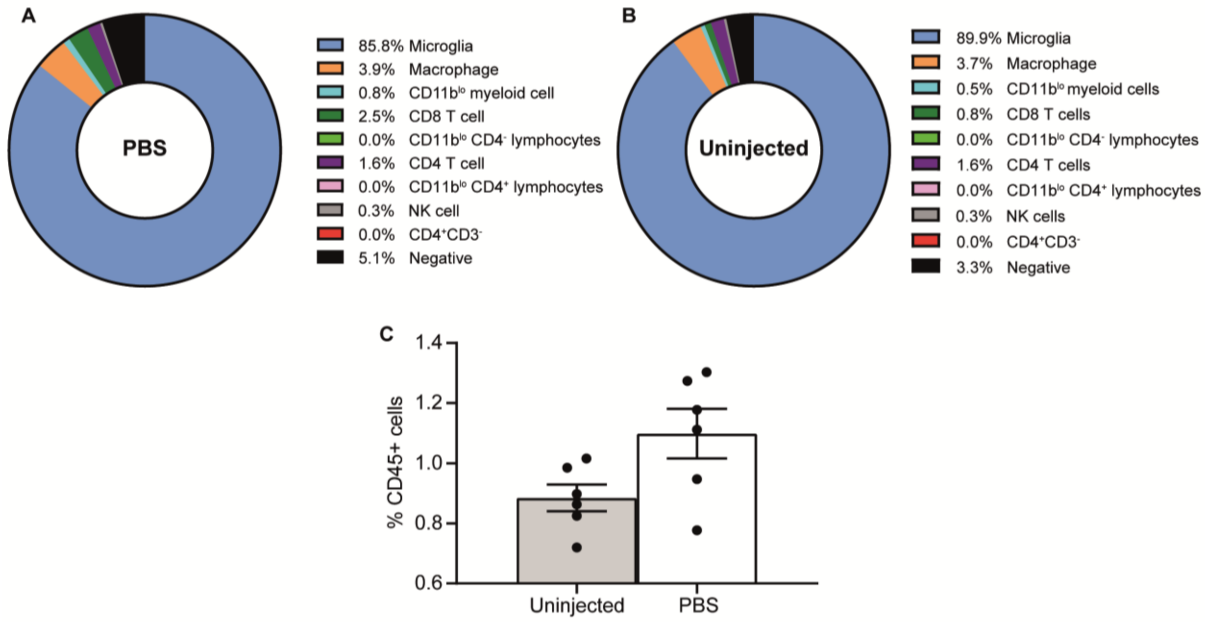

Supplementary figure 4. Leukocyte populations in PBS-injected and uninjected mouse retinae. The mean percentage of each of leukocyte population as a proportion of the total CD45^{+} immune cells (**A**) 28 days after subretinal injection with PBS or (**B**) within uninjected retinae. Assessed using the Cytek Aurora spectral cytometer. Data representative of n=6. (**C**) Percentage of CD45^{+} cells in uninjected baseline and PBS-injected retinae gated to total live cells (±SEM, n=6).

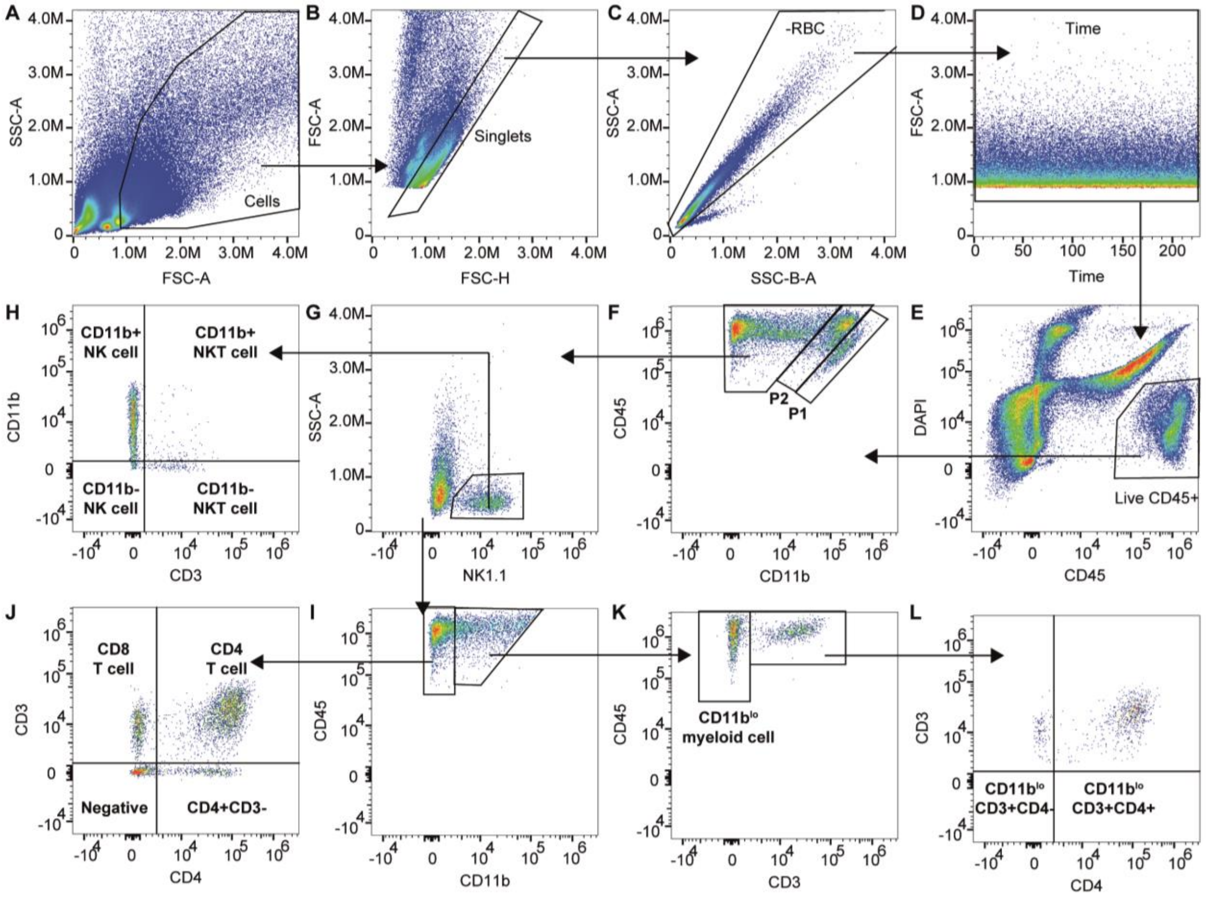

Supplementary figure 5. Gating strategy of an AAV8.CAG.GFP.WPRE-injected mouse retina from Figure 6. The flow cytometry gating strategy of dissociated retinal cells 14 days following injection with an AAV8.CAG.GFP.WPRE vector in C57BL/6J mice in Figure 6 assessed using the Cytek Aurora spectral cytometer. Gating was achieved using FMO controls. The sequential exclusion of (**A**) debris, (**B**) cell doublets, and (**C**) red blood cells. (**D**) Cells that were not acquired in a continuous flow stream were excluded by applying a time gate. (**E**) Live $CD45^{+}$ leukocytes were gated to exclude dead cells and those not of interest in this study. (**F**) Two $CD11b^{hi}$ expressing populations were identified; microglia (P1) and macrophages (P2). (**G**) $NK1.1^{+}$ cells were identified and (**H**) further delineated as $CD11b^{+/-}$ NK and $CD11b^{+/-}$ $CD3^{+}$ NKT cells. (**I**) $CD11b^{-}NK1.1^{-}$ cells were (**J**) identified as $CD3^{+}CD4^{-}$ (CD8 T cells), $CD3^{+}CD4^{+}$ (CD4 T cells), $CD3^{-}$ $CD4^{+}$, and $CD3^{-}CD4^{-}$ (negative). $CD11b^{lo}NK1.1^{-}$ cells were identified as (**K**) $CD3^{-}$ myeloid cells and (**L**) $CD3^{+}$ cells, with the populations $CD11b^{lo}CD3^{+}CD4^{-}$ or $CD11b^{lo}CD3^{+}CD4^{+}$.

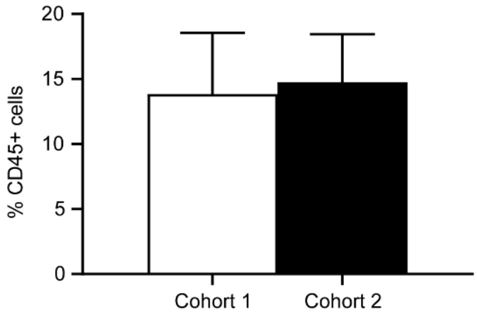

Supplementary figure 6. Repeatability of leukocyte detection using flow cytometric analysis. Percentage of live CD45^{+} cells among all retinal cells in GFP vector-treated retinae from the two experimental cohorts assessed using the Cytek Aurora spectral cytometer (±SEM, n=6).

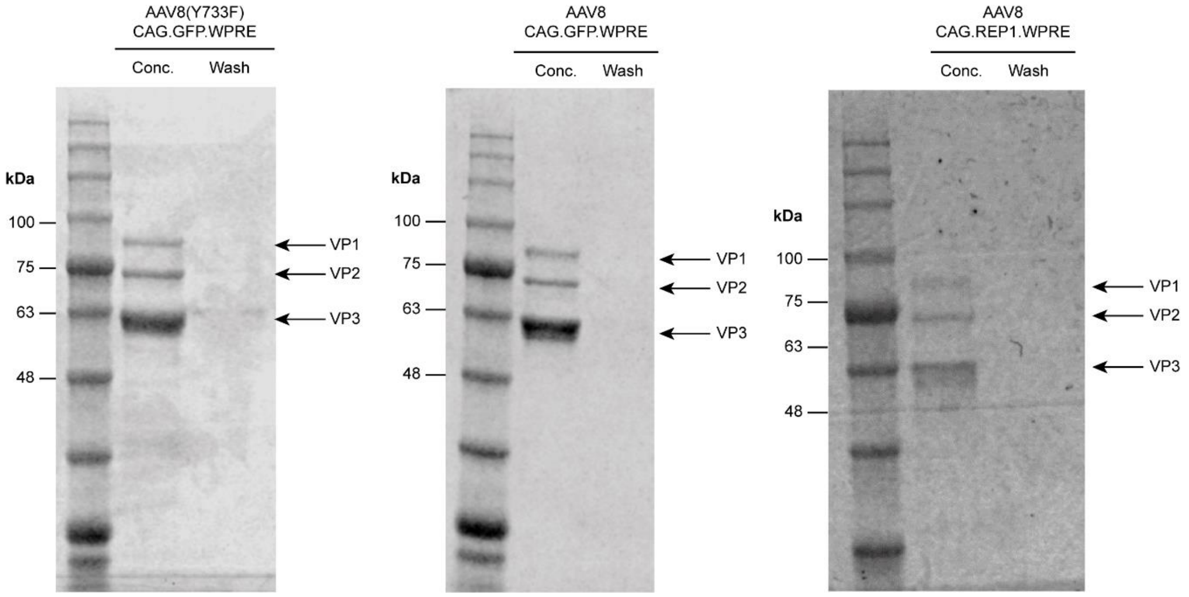

Supplementary figure 7. SDS PAGE of AAV capsid proteins. EZBlue dye stained SDS-PAGE gels of purified preparations of AAV8(Y733F).CAG.GFP.WPRE, AAV8.CAG.GFP.WPRE, and AAV8.CAG.hREP1.WPRE. Images taken on Odyssey Imaging System (LI-COR Biosciences). Bands corresponding to VP1 (87kDa), VP2 (72kDa), and VP3 (62kDa) are indicated in concentrated (conc.) and wash AAV preparations.

Supplementary table 1. Distribution of flow cytometry markers on leukocyte populations.

**Marker**	**Leukocyte population**
CD45	All leukocytes (microglia $CD45^{lo}$, monocytes/macrophages $CD45^{hi}$)
CD11b	Monocytes/macrophages, dendritic cells, granulocytes, natural killer cells, subsets of T and B cells
CD3	T cells, natural killer T cells
CD4	CD4 T cells, subsets of natural killer T cells
CD19	B cells, natural killer–like B cells
CD11c	Dendritic cells
NK1.1	Natural killer cells, natural killer T cells, natural killer–like B cells

Supplementary table 2. Viral genome titers of AAV vectors.

**AAV vector**	**Titer (genome copies/mL)**
AAV8(Y733F).CAG.GFP.WPRE	$3.58x10^{12}$
AAV8.CAG.GFP.WPRE	$1.27x10^{12}$
AAV8.CAG.hREP1.WPRE	$2.01x10^{12}$
